# Cyclodextrin-Derived Porous Liquids Enabled by In Situ Solvation Shell Formation

**DOI:** 10.1021/jacs.6c00992

**Published:** 2026-06-06

**Authors:** Errui Li, Anton S. Pozdeev, Arvind Ganesan, Hongjun Liu, Bo Li, Lilin He, Gergely Nagy, Takeshi Kobayashi, Murillo L. Martins, Yongqiang Cheng, De-en Jiang, Shannon M. Mahurin, Zhenzhen Yang, Sheng Dai

**Affiliations:** † Chemical Sciences Division, 6146 Oak Ridge National Laboratory, Oak Ridge, Tennessee 37831, United States; ‡ Department of Chemistry, Institute for Advanced Materials and Manufacturing, 312344 University of Tennessee, Knoxville, Tennessee 37996, United States; § Department of Chemical and Biomolecular Engineering, 5718 Vanderbilt University, Nashville, Tennessee 37235, United States; ∥ Neutron Scattering Division, Oak Ridge National Laboratory, Oak Ridge, Tennessee 37831, United States; ⊥ U.S. DOE Ames National Laboratory, 1177 Iowa State University, Ames, Iowa 50011, United States

## Abstract

Porous liquids (PLs) represent a unique platform for molecular separations by combining permanent porosity with liquid-phase mobility. However, it remains a formidable challenge to construct and stabilize PLs with sub-5 Å pores using readily available porous host and liquid media. Here, we report the construction of cyclodextrin (CD)-derived PLs enabled by in situ solvation shell formation. The acid–base neutralization reaction between CD and an organic base was leveraged to generate a thin ionic solvation shell around the CD host, effectively liquefying CD and preventing its segregation in the liquid base medium while preserving accessible molecular-scale cavities. Spectroscopic analysis, neutron scattering, density functional theory calculations, and molecular dynamics simulations collectively confirm the structural evolution and existence of abundant internal porosity in PLs. The unique architectures of CD-derived PLs enable highly selective encapsulation of fluorinated alkanes and significantly enhanced uptake of inert gases. This facile and generalizable strategy enables construction of high-quality PLs with engineered ultramicroporosity to facilitate molecular separations.

## Graphical abstract

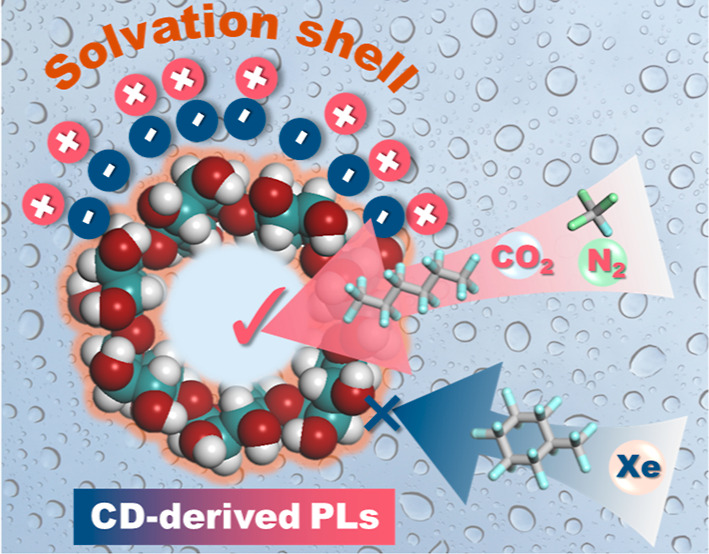

## Introduction

Porous liquids (PLs) represent a unique class of materials that integrate permanent porosity with liquid-phase mobility, enabling selective and enhanced uptake of guest molecules alongside facilitated mass transport.^1–5^ Since their first synthesis in 2015,^6,7^ diverse PLs have been developed to improve separation and catalysis performance in liquid media, circumventing the constraints of traditional structural engineering approaches.^8–11^ PLs are categorized into four types based on the physical state and connectivity of the porous host and liquid phase. Type I PLs consist solely of intrinsically porous components via covalent, ionic, or coordinative modification to form a fluid phase without added solvent.^12–14^ Type II PLs dissolve discrete porous hosts in compatible liquids.^15–20^ Type III PLs involve dispersion of porous solids in sterically hindered and size-excluded solvents.^21–27^ Type IV PLs arise from thermally liquefied metal–organic framework (MOF) glasses that retain porosity in the liquid state.^28–30^ Among these systems, PLs featuring precisely controlled subnanometer porosity are highly desirable for enabling precise molecular sieving during separation.^17,31^ For instance, constructing Type II PLs using cavity-containing discrete molecules, such as porous organic cages (POCs),^7,15–18^ metal–organic polyhedra (MOP),^19,20,32^ and calixarenes,^31^ has shown great promise due to their controllable cavity size at the ultramicropore scale. However, these systems still rely on tedious synthesis of porous hosts, sophisticated surface functionalization, and limited solubility in selected liquid media. It remains a formidable challenge to construct and stabilize PLs with sub-5 Å pores using readily available porous host and liquid media.

Herein, we report a supramolecular strategy to construct cyclodextrin (CD)-derived PLs enabled by in situ solvation shell formation (Figure 1). The acid–base neutralization between hydroxyl groups of CDs and an organic superbase generates a thin ionic solvation shell around each CD host, liquefying the rigid porous matrix, preventing CD aggregation, and greatly enhancing their solubility in liquid basic media, while preserving molecular-scale cavities. Spectroscopy, neutron scattering, and molecular dynamics simulations confirm the structural evolution during liquefaction and retention of internal porosity in PLs. The resulting CD-PLs enable highly selective encapsulation of fluorinated alkanes and enhanced uptake of inert gases, demonstrating efficient molecular sieving at sub-5 Å scales. By integrating renewable, biocompatible CD hosts with engineered sub-5 Å porosity in liquid media, this work establishes a versatile PL platform for molecular separation, selective guest inclusion, and gas storage applications.

**Figure 1:**
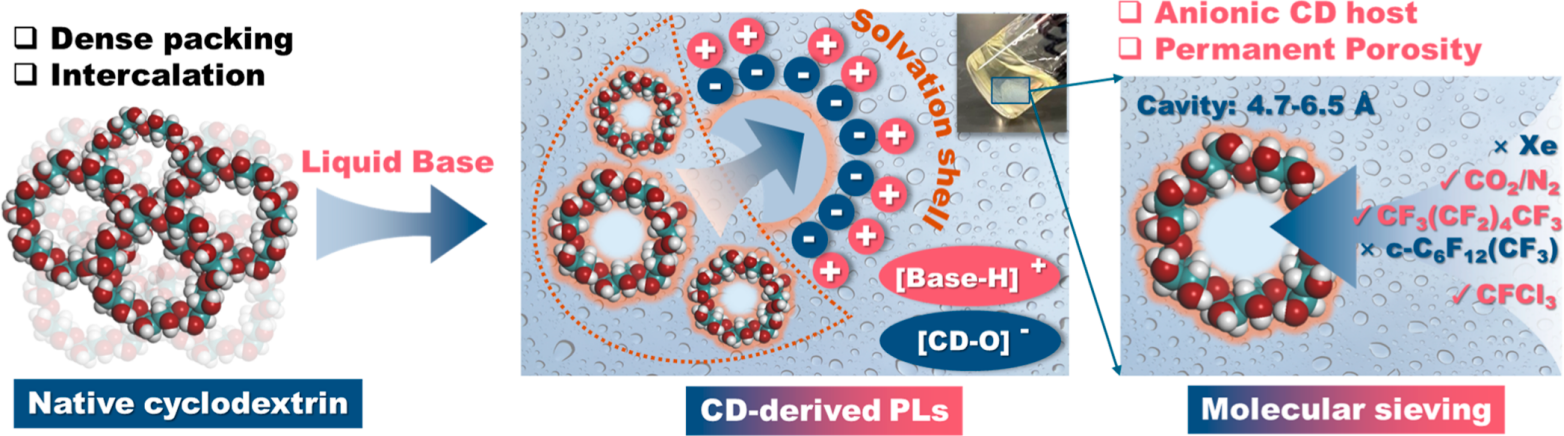
Schematic illustration of CD-derived PL construction via in situ solvation shell formation, enabling the transformation of inaccessible CD cavities into open, accessible pores in liquid media.

## Results and Discussion

Cyclodextrins (CDs) are readily available, biocompatible, and sustainable and feature tunable host–guest chemistry, yet they remain largely underexplored in the design and development of PLs.^33,34^ However, native CDs adopt a cage-type packing in which strong intermolecular hydrogen bonds induce intercalation and block the cavities, rendering them nonporous and unsuitable for direct use in molecular separation (Figure 1).^35^ CDs can be converted into channel-type packings via guest-induced reprecipitation processes.^36^ In these cases, the intrinsic cavities of CDs are occupied by guest molecules, and high-temperature removal of the guests causes the packing to revert to a nonporous structure. Alternatively, rigid CD-based frameworks have been constructed to preserve accessible porosity through cross-linking or metal coordination.^37^ However, integrating the intrinsic cavities of CDs with liquid-phase flowability remains a formidable challenge.

The key to synthesizing high-quality Type II PLs lies in enhancing the solubility of the porous host in size-exclusive liquid media. Conventional approaches typically require complex chemical modifications or de novo synthesis to improve the solubility of organic or coordination cages.^7,20^ Here, we use an in situ solvation shell formation approach to construct Type II PL from readily available CDs and liquid components (organic base) without any functionalization treatment. αCD, a rigid, shape-persistent macrocycle with well-defined intrinsic cavities (4.7–5.2 Å) and peripheral hydroxyl groups, was selected as the porous host due to its ability to retain molecular-scale porosity in the bulk liquid state when crystallization and aggregation are suppressed. The hydroxyl groups on αCD act as weak Brønsted acids (p*K*_a_ = 12–13.5 in H_2_O),^38^ providing chemically active sites for acid–base interactions. Additionally, 1,8-diazabicyclo[5.4.0]­undec-7-ene (DBU) with a high proton affinity (p*K*_a_ = 24.3 in acetonitrile) was deployed as a basic and liquid counterpart (Figure 2A),^39^ due to its potential capability to deprotonate hydroxyl groups of αCD and forming stable ionic adducts in situ. Importantly, DBU is sterically hindered and has a nonplanar structure (molecular size: 5.4 × 7.6 × 10.2 Å^3^, Figure S1), which limits its ability to occupy the internal cavities of αCD, thereby preserving accessible porosity in the resulting liquid phase. This facile and rational pairing of αCD and DBU represents a chemically straightforward and potentially scalable route to PLs construction.

**Figure 2:**
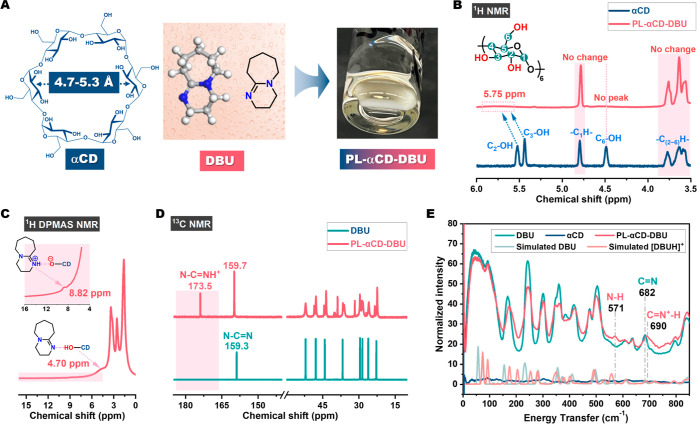
(A) Chemical structures of αCD, DBU, and optical image of PL-αCD-DBU. (B) ^1^H NMR spectra of αCD and PL-αCD-DBU. (C) ^1^H DPMAS NMR spectrum of PL-αCD-DBU. (D) Solvent-free ^13^C NMR spectra of DBU and PL-αCD-DBU. (E) INS spectra of DBU, αCD, and PL-αCD-DBU.

αCD and DBU were mixed in a molar ratio of 1:18 (equimolar ratios of hydroxyl groups in αCD to DBU) and reacted at 55 °C without catalysts or solvents. Upon cooling to ambient temperature, a transparent and stable liquid phase was formed (denoted as PL-αCD-DBU). Its liquid behavior was determined by the oscillatory shear measurements at 298 K, as the loss modulus (*G*″) is consistently higher than the storage modulus (*G*′), meanwhile showing an average viscosity of 113 Pa·s (Figure S2). Moreover, viscosity decreases with increasing temperature (Figure S3), consistent with ionic liquid-like behavior. Structural evolutions during the solvation process were characterized by nuclear magnetic resonance (NMR), Fourier-transform infrared (FT-IR) spectroscopy, and inelastic neutron scattering (INS). As shown in the ^1^H NMR spectra (Figure 2B), upon mixing αCD with DBU, the hydroxy protons on αCD (−OH–C_2_ and −OH–C_3_, originally at 5.52 and 5.44 ppm) downfield-shifted to 5.75 ppm and formed a broad signal. Another hydroxy proton of C_6_–OH at 4.49 ppm disappeared, suggesting deprotonation reaction occurred. Meanwhile, the proton signal for the −CH– and –CH_2_– groups of αCD (4.79 and 3.77–3.25 ppm) remained largely unchanged, indicating intact skeleton of αCD units. Solid-state ^1^H direct-polarization magic-angle spinning (DPMAS) NMR was performed to eliminate solvent effects (Figure 2C). The signal at 4.70 ppm is assigned to –OH groups involved in hydrogen bonding with DBU,^40^ while the signal at 8.82 ppm corresponds to the protonated DBU cation ([DBUH]^+^).^41^ The coexistence of these two signals directly evidences a mixed interaction mode, in which hydrogen-bonded αCD-DBU adducts and proton-transferred species are simultaneously present.

The occurrence of deprotonation was further confirmed by solvent-free ^13^C NMR spectra (Figure 2D). To observe more pronounced spectral changes, a higher molar ratio of αCD to DBU with 1:36 was employed. Compared with neat DBU, the PL-αCD-DBU complex exhibited two sets of carbon signals. Notably, besides the signal of quaternary carbon (N–C=N) in free DBU (159 ppm), the protonated DBU cation formation was confirmed by the new peak at 173.5 ppm (N–C=NH^+^). The corresponding FT-IR spectra revealed that the broad O–H stretching band at 3288 cm^–1^ progressively weakened and nearly disappeared after mixing with DBU, accompanied by the appearance of an N–H stretching band at 3347 cm^–1^ (Figure S4). Additionally, the methylene C–H stretching of DBU at 2846 cm^–1^ exhibited a blue-shift, while the C=N stretching near 1610 cm^–1^ showed a red-shift, consistent with the protonated DBU cation formation.^42^ Thermogravimetric analysis (TGA) showed that PL-αCD-DBU began to lose weight at 98.2 °C due to the release of free or protonated DBU species, followed by decomposition of the cyclodextrin framework above 200 °C (Figure S5). These changes in the fingerprint region further corroborate the generation of anionic pairs. INS spectra further corroborated the deprotonation process, providing enhanced sensitivity to N–H and low-frequency modes crucial for identifying proton transfer (Figure 2E). Compared with pristine DBU, most vibrational features of PL-αCD-DBU appear at essentially the same positions, while the contribution of αCD to the overall INS intensity is negligible. Notably, a new band emerged at 571 cm^–1^ in PL-αCD-DBU, which is assigned to the N–H scissoring vibration, indicating protonation of DBU. Meanwhile, the C=N stretching mode of DBU shifted from 682 to 690 cm^–1^ upon protonation. The observed band broadening originated from the coexistence of neutral DBU and protonated [DBUH]^+^ species. The corresponding peak assignments were supported by simulated phonon bands of neutral DBU and protonated DBU (Figure S6).

To elucidate the DBU−αCD interactions at peripheral rim hydroxyls, we evaluated formation energies for hydrogen-bonded versus proton-transfer adducts at the C_2_, C_3_ (secondary) and C_6_ (primary) positions using TPSS/def2-SVPD and TPSSh/def2-SVPD levels of theory, reporting Δ*G* values (Figure 3). At the secondary sites, neutral H-bonding is favored over proton transfer by ΔΔ*G* = 5–10 kJ/mol (Table S1). The optimized neutral complexes display N···H distances of 1.62–1.66 Å with O–H covalent bonds of 1.02–1.04 Å (Table S2). In contrast, at the primary C_6_ site, proton transfer is slightly preferred (ΔΔ*G* = −2.3 kJ/mol relative to the H-bonded adduct at TPSSh/def2-SVPD), forming a short N–H bond of 1.15 Å and an elongated O···H bond of 1.39 Å, typical for a [DBUH]^+^···O^–^ contact ion pair. Across sites and functionals, the absolute formation free energies vary from around −26 to −44 kJ/mol, indicating strong interaction. As a cavity-free model system, glucose-DBU at C_6_ forms only a weak neutral H bond (Δ*E* ≈ −62 kJ/mol; ΔΔ*G* ≈ +1–3 kJ/mol), highlighting the macrocyclic environment’s role in stabilizing the DBU-CD adducts. The absence of a proton-transfer minimum further indicates that such transfer is strongly favored within the cyclodextrin cavity.

**Figure 3:**
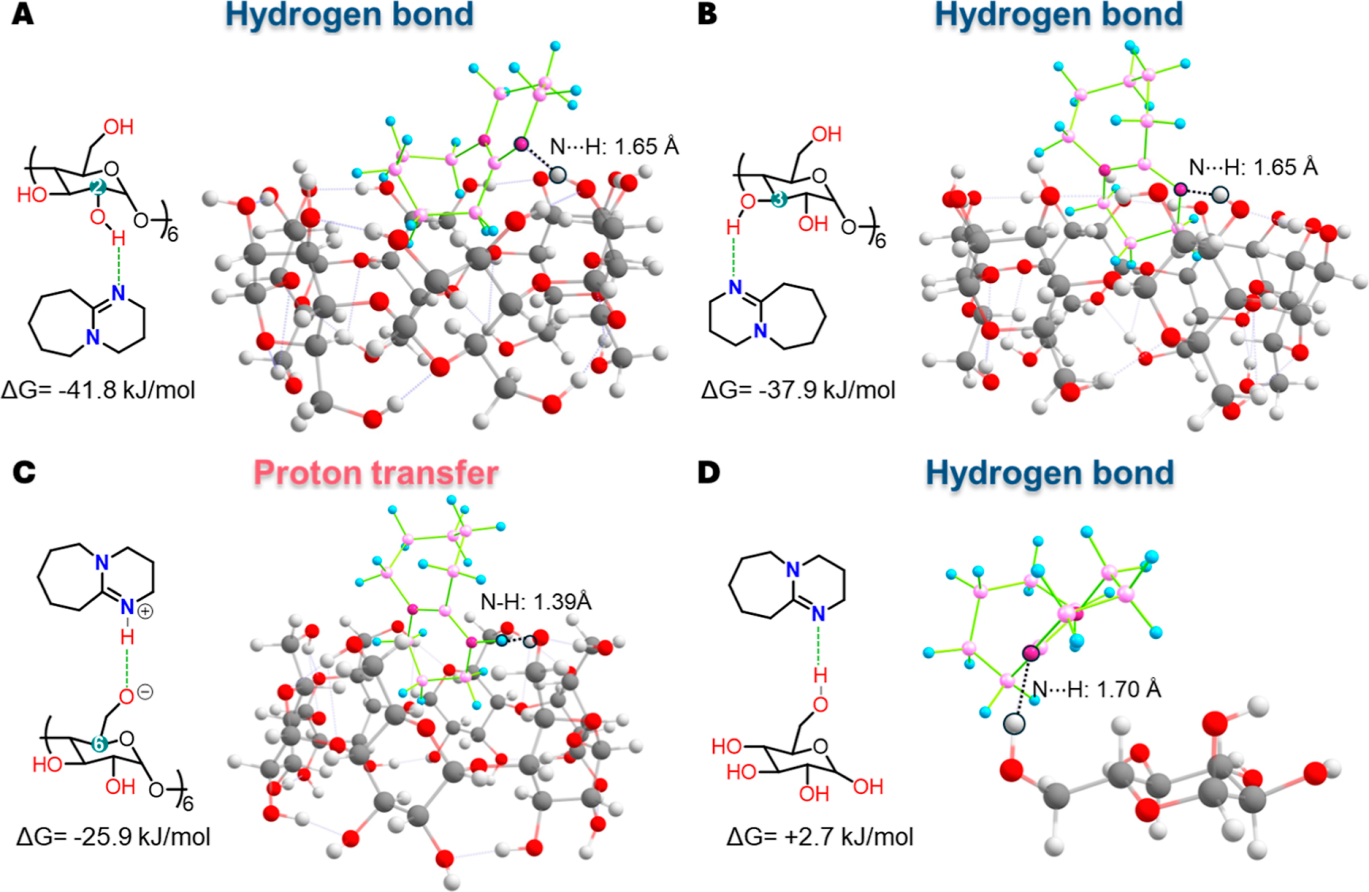
(A–C) Comparison of formation free energy between αCD and DBU at different –OH sites. (D) Formation free energy between glucose and DBU. Δ*G* values were obtained at the DFT TPSSh/def2-SVPD level for 298.15 K.

Our calculations suggest that hydrogen bonding likely dominates the interaction between αCD and DBU. Proton transfer occurs preferentially at the primary C_6_ hydroxyl groups, which is more favorable than at the secondary C_2_ and C_3_ sites (tend to remain H-bonded). This site-selective proton transfer provides a direct explanation for the experimental spectroscopic signatures (NMR, FT-IR, INS) of protonated DBU, as even a limited population of C_6_-associated ion pairs can dominate these signals due to their strong vibrational and electronic responses. Therefore, the αCD-DBU system can be described as a spatially heterogeneous and dynamically equilibrated mixture, rather than a uniformly protonated structure. The macrocyclic framework of αCD therefore promotes localized charge stabilization that helps disperse the host molecules in liquid DBU while keeping their internal cavities accessible, which is expected behavior for Type II PLs.

The real-space porosity nature of PL-αCD-DBU was then investigated using small-angle neutron scattering (SANS), a technique highly sensitive to light elements and well-suited for probing nanoscale structural features. The SANS profile of pristine solid αCD exhibited distinct diffraction peaks at 0.37, 0.87, and 1.59 Å^–1^ in the high-q region (corresponding to real-space distances of 16.9, 7.2, and 3.9 Å, respectively, Figure 4A). These features are characteristic of the periodic motifs within the αCD structure, corresponding to the outer periphery, torus height, and intrinsic microporosity (Figure S7). In the low-q region, the sharp slope of αCD suggested its well-defined crystalline domains. Upon formation of PL-αCD-DBU, the SANS pattern underwent substantial changes that collectively indicated dissolution of αCD. Most of the αCD diffraction peaks disappeared, while the characteristic short-range ordering peak of neat DBU at 1.18 Å^–1^ became strongly attenuated, reflecting loss of its molecular correlations (Figure S1). Despite this overall reduction in order, several αCD-derived peaks including 0.40, 0.87, and 1.58 Å^–1^ remained detectable, signifying retention of local structural integrity and suggesting that microporosity persists in the liquid environment. Additionally, the low-q region became flat and nearly indistinguishable from pure DBU, consistent with homogeneous molecular dispersion rather than aggregation or domain formation.

**Figure 4:**
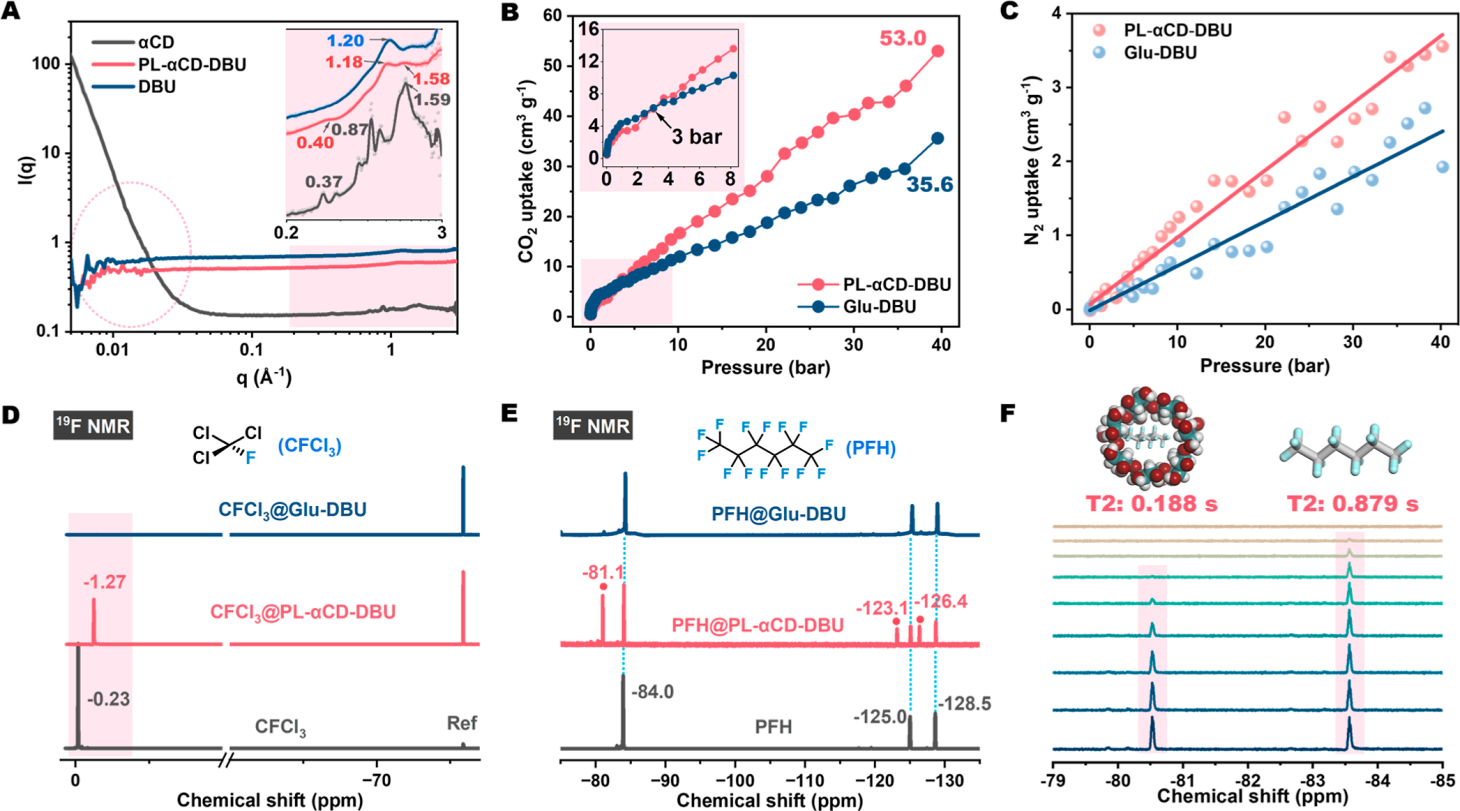
(A) SANS profiles of αCD, DBU, and PL-αCD-DBU. (B) CO_2_ and (C) N_2_ uptake isotherms of Glu-DBU and PL-αCD-DBU at 298 K. (D) Solvent-free ^19^F NMR spectra of CFCl_3_, CFCl_3_@PL-αCD-DBU, and CFCl_3_@Glu-DBU, with sealed trifluoroacetic acid as the reference solvent (peak labeled as Ref). (E) Solvent-free ^19^F NMR spectra of PFH, PFH@PL-αCD-DBU, and PFH@Glu-DBU. (F) ^19^F NMR spectra for the T2 relaxation measurement of PFH@PL-αCD-DBU.

The intrinsic porosity in PL-αCD-DBU was further evaluated using gas sorption isotherms and fluorinated alkane encapsulation experiments. For a detailed comparison and to exclude potential effects arising from chemical interactions, a control mixture of DBU and glucose, representing the monomeric unit of CD without cavity, was prepared under identical conditions (denoted as Glu-DBU) (Figures S8–S14). The pressure-dependent CO_2_ uptake isotherms of Glu-DBU and PL-αCD-DBU were collected at 298 K. Below 0.5 bar, both systems exhibited a sharp increase in CO_2_ uptake, attributed to the presence of multiple oxygenate sites and free DBU molecules that interact favorably with CO_2_. At CO_2_ pressure below 3 bar, Glu-DBU and PL-αCD-DBU exhibited similar CO_2_ uptake behavior. However, within the high-pressure region, PL-αCD-DBU demonstrated a marked enhancement in CO_2_ adsorption. At a CO_2_ pressure of 40 bar (Figure 4B), PL-αCD-DBU achieved a CO_2_ uptake of 53.0 cm^3^ g^–1^, representing a 48.8% increase compared to Glu-DBU (35.6 cm^3^ g^–1^). Then N_2_ was used as a probe molecule to further exclude the influence from chemisorption. In the N_2_ uptake isotherms (Figure 4C), PL-αCD-DBU consistently exhibited a higher N_2_ uptake than Glu-DBU across the entire pressure range (up to 40 bar), indicating the presence of accessible internal cavity in PL-αCD-DBU that persists in the liquid phase. When Xe gas was used as a probe with a bigger kinetic diameter of 3.9 Å, the ^129^Xe NMR spectra of both PL-αCD-DBU and Glu-DBU exhibited noticeable downfield shifts relative to pure DBU (Figure S15). Given that downfield shifts are generally associated with restricted environments and enhanced host–guest interactions, these results indicated that Xe experiences a more confined microenvironment in both systems. For Glu-DBU, a shift of 4.65 ppm was observed, because the ions exist primarily as localized contact ion pairs with spatially confined charges. In the PL-αCD-DBU system, an even greater downfield shift was obtained, reflecting a more sterically constrained microenvironment.

Considering the hydrophobic cavities of CDs and their ionized external surface, hydrophobic probe molecules were selected to assess guest encapsulation. The binding effect of CFCl_3_ was investigated first through the vapor diffusion method. Solvent-free ^19^F NMR spectra were then recorded to distinguish free and encapsulated species (Figure 4D). Compared with pure CFCl_3_, the signal showed an upfield shift from −0.23 ppm to −1.27 ppm upon mixing with PL-αCD-DBU, whereas a negligible signal of CFCl_3_ was observed in the nonporous Glu-DBU system, indicating the essential role of the αCD cavity to trap CFCl_3_ during the diffusion procedure. Additionally, perfluorohexane (PFH) with lower polarity showed identical resonances in Glu-DBU to those of pure PFH (Figure 4E). Comparatively, PFH in PL-αCD-DBU produced an additional set of downfield-shifted peaks alongside the free PFH peaks, confirming the encapsulation of PFH within αCD cavities.

The dynamic motion of PFH in PL-αCD-DBU was further monitored via ^19^F NMR relaxation experiments. T2 relaxation time, calculated by fitting the decay curves (Figures 4F and S16), revealed slower dynamics for encapsulated PFH. The peak at −81.1 ppm showed a shorter T_2_ (0.188 s) compared to the free PFH resonance at −84.1 ppm (0.879 s). These results demonstrate that confinement within the αCD cavity imposes restricted motion and enhanced dipole–dipole interactions.^43^ Collectively, these observations confirm the presence of molecular-scale voids in PL-αCD-DBU. In contrast, when a larger probe molecule, perfluoromethylcyclohexane (PFMCyH) (Figure S17), was tested, only a single set of ^19^F resonances was detected in both Glu-DBU and PL-αCD-DBU systems, identical to pure PFMCH, highlighting the capability of PL-αCD-DBU to separate fluorinated alkanes with structural similarity but subtle size difference (Figure S18).

Molecular dynamics (MD) simulations were conducted to understand the structural characteristics of PL systems and the accessible porosity inside. Based on the experimental structure information, PL models containing 20 negatively charged αCD, 240 DBU, and 120 protonated DBU were deployed and placed in a cubic and periodic simulation box under the *NPT* ensemble at 298 K and 1 bar. As shown in the representative molecular dynamics snapshot of PL-αCD-DBU (Figure 5A), no aggregation of the charged porous host ([αCD-O]^−^) was observed throughout the simulation. The protonated DBU cations are preferentially localized near the αCD portals, forming a stable solvation shell. Meanwhile, the negatively charged αCD molecules remained well-dispersed and were surrounded by free DBU molecules, with no evidence of DBU penetration into the intrinsic cavities. To further verify cavity preservation, radial distribution functions (RDFs) between the αCD geometry center and DBU/[DBUH]^+^ were calculated (Figure 5B). For neutral DBU, negligible molecules were detected within a 6 Å probe radius from the αCD center. While with a 2.5 Å probing distance, an average of 0.29 [DBUH]^+^ molecules were observed, consistent with [DBUH]^+^ localization at the αCD periphery. This distribution is consistent with a Derjaguin–Landau–Verwey–Overbeek (DLVO)-like stabilization modified by specific ion adsorption.^44,45^ Adsorbed [DBUH]^+^ forms a positively charged corona around each [αCD-O]^−^, generating electrostatic double-layer repulsion that offsets van der Waals attraction and prevents [αCD-O]^−^–[αCD-O]^−^ aggregation, while steric and solvation effects from the [DBUH]^+^ shell further reduce effective attraction and help preserve intrinsic cavities.

**Figure 5:**
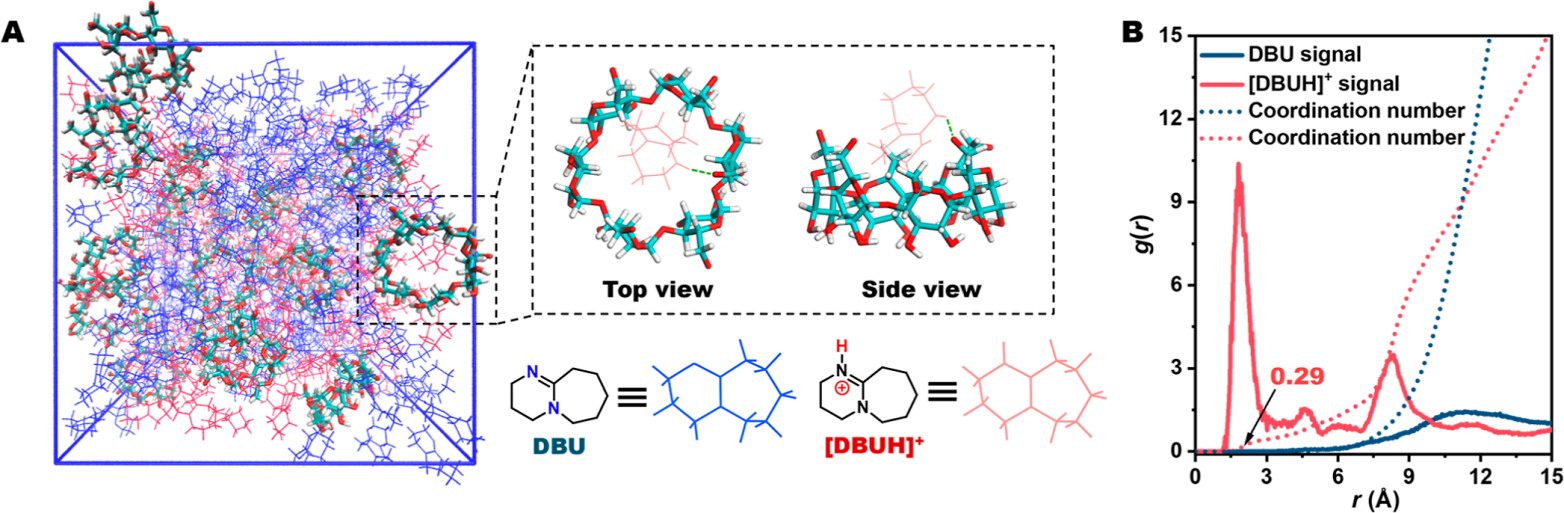
(A) MD-simulated structure of PL-αCD-DBU. (B) RDFs from the geometry center of αCD to DBU and [DBUH]^+^.

The as-developed facile solvation shell formation approach was extended to diverse organic base media and CD analogs with larger cavity sizes. Transparent liquids were obtained for additional CD–superbase pairs, including αCD-MTBD (7-methyl-1,5,7-triazabicyclo[4.4.0]­dec-5-ene, MTBD, Figure S19), βCD-DBU, and γCD-DBU (Figures 6A,B and S20). These transformations were proved by liquid NMR and FT-IR (Figures S15–S36). Specifically, solvent-free ^13^C NMR spectra of the products showed two sets of organic base signals (Figure 6C,D), similar to PL-αCD-DBU (Figure 2C). The proton transfer between CD and MTBD was further evidenced by INS spectra (Figure 6E). Upon protonation, the C=N stretching mode of MTBD shifted from 701 to 708 cm^–1^, accompanied by the disappearance of several C–H scissoring/twisting/wagging modes of MTBD in the low-energy region (Figure S37). Cavity preservation was probed by SANS. αCD- and βCD-based PLs retained the characteristic CD cavity peak in the liquid phase (Figure 6F,G), whereas γCD-DBU lost porosity due to the larger cavity permitting DBU penetration (Figure S38). This was corroborated by PFH encapsulation experiments. PFH@PL-αCD-MTBD and PFH@PL-βCD-DBU displayed two sets of ^19^F signals corresponding to free and confined PFH, whereas PFH@γCD-DBU showed a single set of signals identical to pure PFH (Figure 6H). For a larger probe, all systems exhibited one set of ^19^F signals identical to pure PFMCyH (Figure S39), indicating that α- and β-CD cavities cannot accommodate this bulky guest and that γCD lacks accessible porosity (Figures S40 and S41). This observation highlights a critical size-matching requirement: the host cavity must be sufficiently large to accommodate target guest molecules, yet sufficiently restricted to exclude solvent or base components that would otherwise occupy the cavity and eliminate accessible porosity. In the γCD-DBU system, the relatively large cavity of γCD (7.5–8.3 Å) permits DBU penetration (10.23 × 7.65 × 5.39 Å^3^), resulting in partial occupation of the intrinsic free volume and loss of porosity. Thus, this system represents a boundary case that defines the upper size limit of cavity dimensions for preserving permanent porosity. More importantly, this result underscores the necessity of steric hindrance control as a central design criterion, ensuring effective exclusion of competing species while maintaining accessibility to target guests.

**Figure 6:**
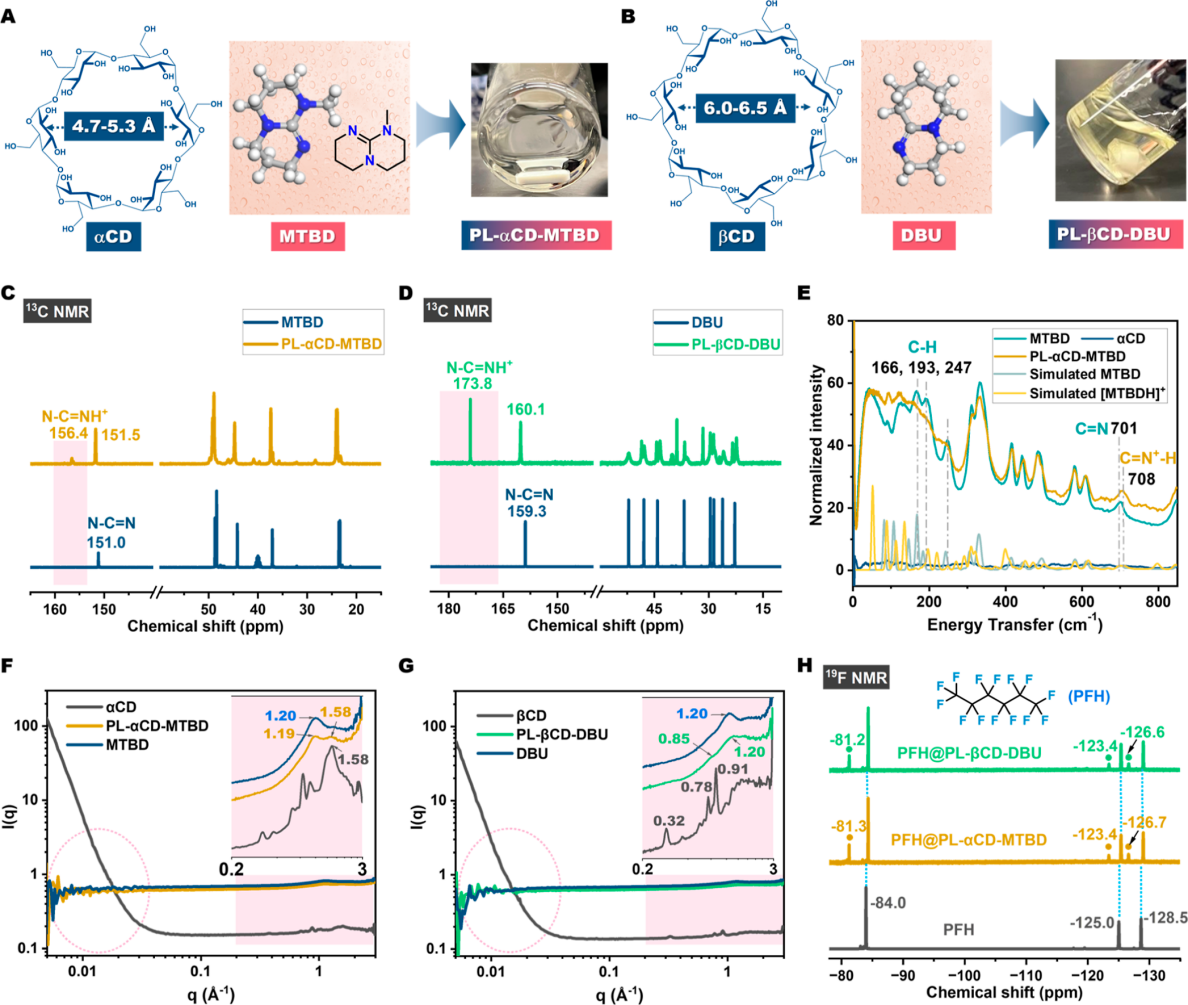
(A) Chemical structures of αCD, MTBD, and optical image of PL-αCD-MTBD. (B) Chemical structures of βCD and optical image of PL-βCD-DBU. (C) ^13^C NMR spectra of MTBD and PL-αCD-MTBD. (D) ^13^C NMR spectra of DBU and PL-βCD-DBU. (E) INS spectra of MTBD, αCD, and PL-αCD-MTBD. (F) SANS profiles of αCD, MTBD, and PL-αCD-MTBD. (G) SANS profiles of βCD, DBU, and PL-βCD-DBU. (H) ^19^F NMR spectra of PFH, PFH@PL-αCD-MTBD, and PFH@PL-βCD-DBU.

In conclusion, this work establishes a supramolecular acid–base neutralization strategy to transform CDs into flowable PLs while retaining their intrinsic molecular cavities. The in situ formation of ionic solvation shells effectively suppresses aggregation of CDs, yielding stable, homogeneous liquids with permanent and tunable subnanometer porosities. Spectroscopy analysis, neutron scattering detection, and small guest molecule trapping experiments collectively confirm that the cavities of CDs remain accessible in the liquid state, enabling selective guest inclusion and molecular sieving behavior. Notably, the synergistic use of mixed-solvent systems is expected to offer a versatile platform for decoupling the long-standing trade-off between porosity and mobility, thereby potentially enabling improved transport efficiency without sacrificing selectivity. This approach demonstrates a general route for integrating readily available macrocyclic hosts into functional PLs, bridging molecular precision with fluid processability.

## Supplementary Material



## Notes

Information on chemicals, characterization, synthesis of PLs, theoretical calculation method, and supplementary figures (PDF)
