# Supplementary material for: Cyclodextrin-Derived Porous Liquids Enabled by In Situ Solvation Shell Formation
Source: J Am Chem Soc. 2026 Jun 6;148(23):23707–15. doi: 10.1021/jacs.6c00992 (PMC13281529; doi:10.1021/jacs.6c00992)
Supplement: Supplementary file 1 [file ja6c00992_si_001.pdf]

Supporting Information

Cyclodextrin-Derived Porous Liquids Enabled by In Situ Solvation Shell Formation

Errui Li,[1,2] Anton Pozdeev,[3] Arvind Ganesan,[1] Hongjun Liu,[3] Bo Li,[3] Lilin He,[4] Gergely Nagy,[4] Takeshi Kobayashi,[5] Murillo L. Martins,[4] Yongqiang Cheng,[4] De-en Jiang,[3] Shannon M. Mahurin,[1] Zhenzhen Yang,[,1] and Sheng Dai*[,1,2]*

[1] Chemical Sciences Division, Oak Ridge National Laboratory, Oak Ridge, TN 37831, USA

[2] Department of Chemistry, University of Tennessee, Knoxville, TN 37996, USA

[3] Department of Chemical and Biomolecular Engineering, Vanderbilt University, Nashville, TN 37235, USA

[4] Neutron Scattering Division, Oak Ridge National Laboratory, Oak Ridge, TN 37831, USA

[5] U.S. DOE Ames National Laboratory, Iowa State University, Ames, IA 50011, USA

E-mail: yangz3@ornl.gov; dais@ornl.gov

1. Experimental Procedures

1.1 Materials

αCD, βCD γCD, glucose, 1,8-diazabicyclo[5.4.0]undec-7-ene (DBU), 7-methyl-1,5,7-triazabicyclo[4.4.0]dec-5-ene (MTBD), trichlorofluoromethane ($CFCl_3$), perfluorohexane (PFH), perfluoromethylcyclohexane (PFMCyH), and solvents (DMSO, trifluoroacetic acid) were purchased from commercial sources (TCI, Fisher scientific, Eckert & Ziegler, and Sigma-Aldrich) and were used without further purification unless indicated otherwise.

1.2 Characterization

Rheological properties were tested on the AR-G2 instrumentation by using steel plate geometry with a 25 mm diameter plate. In the oscillation-dependent modulus measurements, the temperature was fixed at 25°C. For viscosity-temperature measurements, the angular frequency was fixed at 10 rad/s and the ramps at 5 °C min^{-1}.

Liquid ^{1}H NMR, ^{13}C NMR and ^{19}F NMR spectra were recorded on Bruker 400 MHz spectrometer at ambient temperature. ^{1}H NMR chemical shifts were referenced to the residual solvent of DMSO-d_6. For solvent-free ^{13}C NMR, a sealed capillary containing DMSO was used for field locking and chemical shift referencing. For solvent-free ^{19}F NMR, a sealed capillary containing DMSO-d_6 was used for field locking, and trifluoroacetic acid served as the external reference for chemical shifts.

Solid-state ^{1}H direct-polarization magic-angle spinning (DPMAS) NMR experiments were performed on a Bruker NEO spectrometer equipped with a 2.5 mm triple resonance MAS probe, operating at 14.1 T. The sample was packed in the zirconia MAS rotor. ^{1}H DP spin–echo experiments were performed at 20 kHz. ^{1}H chemical shift is reported using the δ scale, referenced to tetramethylsilane (TMS) at 0 ppm.

Fourier-transform infrared (FT-IR) characterization of the samples was collected on a Nicolet Nexus iS50 FTIR instrument in the spectrum range from 4000–500 cm^{-1}.

Thermogravimetric analysis (TGA) measurements were taken under N_2 atmosphere with a ramping rate of 10 °C min^{-1} from 25−800 °C by using TGA Q50 thermogravimetric analyzer.

Inelastic neutron scattering (INS): Neutron vibrational spectra were collected at the VISION beamline of the Spallation Neutron Source, Oak Ridge National Laboratory. VISION is an indirect-geometry, crystal-analyzer neutron spectrometer designed for high-resolution vibrational spectroscopy. It provides high neutron flux for broad-band measurements of molecular and lattice dynamics, particularly on chemical systems and processes. The analysis of the measured VISION and assignment of peaks were assisted by computer simulations. For all the molecular models, geometry optimization and vibrational analysis were first performed via Gaussian, with B3LYP/6-311++G(d,p) level of theory.[1] The INS spectra of the molecules were then simulated by OCLIMAX, using the normal mode frequencies and eigenvectors solved from the molecular Hessian matrices.[2]

Small angle neutron scattering (SANS) data were collected at the EQ-SANS instrument, at the Spallation Neutron Source (SNS) of the Oak Ridge National Laboratory, Oak Ridge, TN. The instrument configurations were set to cover a q-range of $0.005 < q < 3$ Å^{-1}.[3, 4] The data was

corrected for detector sensitivity, background scattering and transmission and was azimuthally averaged to get 1D curves, which was shown in a double logarithmic graph in Figures 4A ,6F, 6G and S23.

High pressure gas sorption isotherms (40 bar for CO_2 and N_2) were obtained with HPVA II high-pressure volumetric analyzer (Micromeritics). Samples were degassed at 35 °C for 5 h.

^{19}F NMR spectra for T_2 relaxometry measurements were collected on Varian INOVA 500. T_2 relaxation time was determined by analyzing integral decay curves and fitting the magnetization decay (signal intensities: peak heights and integral values) to a mono-exponential function in MestReNova software.

$$M(t) = B * e^{-Ft} \qquad (2)$$

where B is the magnetization at time zero and F=1/T_2.

1.3 Synthesis

Synthesis of PL-αCD-DBU: Solid powder of αCD (243 mg, 0.25 mmol, 1.0 eq.) and DBU (685 mg, 4.5 mmol, 18.0 eq.) were mixed and stirred at 55 °C overnight to obtain transparent liquid.

Synthesis of Glu-DBU: Solid powder of glucose (90 mg, 0.5 mmol, 1.0 eq.) and DBU (380.5 mg, 2.5 mmol, 5.0 eq.) were mixed and stirred at 55 °C overnight to obtain transparent liquid.

Synthesis of PL-αCD-MTBD: Solid powder of αCD (243 mg, 0.25 mmol, 1.0 eq.) and MTBD (689 mg, 4.5 mmol, 18.0 eq.) were mixed and stirred at 55 °C overnight to obtain transparent liquid.

Synthesis of PL-βCD-DBU: Solid powder of βCD (283 mg, 0.25 mmol, 1.0 eq.) and DBU (799 mg, 5.25 mmol, 21.0 eq.) were mixed and stirred at 55 °C overnight to obtain transparent liquid.

Synthesis of γCD-DBU: Solid powder of γCD (324 mg, 0.25 mmol, 1.0 eq.) and DBU (913 mg, 6.0 mmol, 24.0 eq.) were mixed and stirred at 55 °C overnight to obtain transparent liquid.

1.4 DFT calculations

Model systems and binding motifs: we modeled 1:1 adducts between αCD and DBU at the three nonequivalent hydroxyl positions: secondary-side C2 and C3, and primary-side C6. For each site we optimized the following motifs:

- Neutral H-bonded complex: αCD-OH···N(DBU), no proton transfer.
- H-transfer adduct: αCD-O^-···H–N^+(DBU), i.e., a contact ion pair.
- Additionally, as a cavity-free test, we computed the glucose–DBU C6-complex.

Electronic structure: all geometries were first optimized with TPSS-D4/def2-SVPD.[5-9] Harmonic frequencies were computed at this level of theory to confirm that the stationary points are minima, i.e., no imaginary frequencies, and to obtain thermochemical corrections. Then, each structure was re-optimized with TPSSh-D4/def2-SVPD[10] to the nearest local minimum. Frequencies were not recomputed at TPSSh. Consequently:

- Reported ΔG values labeled “TPSS/def2-SVPD” are pure TPSS free energies (TPSS electronic energies plus TPSS thermochemical corrections from TPSS frequencies).

- Reported ΔG values labeled as "TPSSh/def2-SVPD" are composite free energies, $\Delta G^{\mathrm{TPSSh}} = E_{\mathrm{elec}}^{\mathrm{TPSSh}} + \Delta G_{\mathrm{corr}}^{\mathrm{TPSS}}$.

Solvation was modeled with CPCM (relative permittivity ε = 12.0, refractive index n = 1.523)[11] to emulate a base-rich organic environment. Diffuse functions were employed to stabilize charge-separated ion pairs in a low-dielectric medium. All calculations used defgrid3, VeryTightSCF, and TightOpt settings as implemented in ORCA 6.0.1.[12]

Molecular Dynamics Simulations: Classical molecular dynamics simulations were carried out with the GROMACS package.[13] PL-αCD-DBU composed of 20 αCD polyanions with deprotonated primary C6 hydroxyl sites, 240 DBU molecules, and 120 $DBUH^{+}$ cations were modeled with the general Amber force field (GAFF).[14] The structure of the whole system was first geometry optimized, then followed by at least 100 ns equilibration in NPT ensemble at 298 K and 1 bar. Another 100 ns NVT simulation was performed for production.

2. Results and Discussion

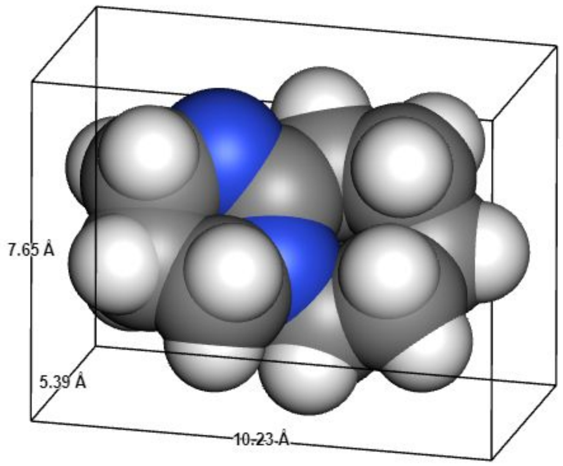

Figure S1. Space-filling model of DBU showing the molecular dimensions (10.23 × 7.65 × 5.39 $Å^3$).

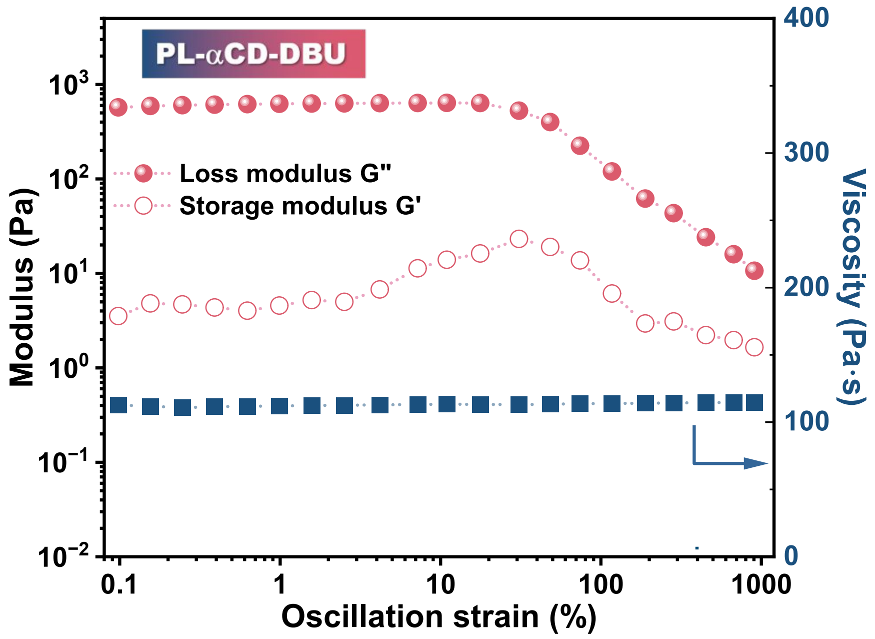

Figure S2. Oscillation-dependent modulus plots and viscosity of PL-αCD-DBU.

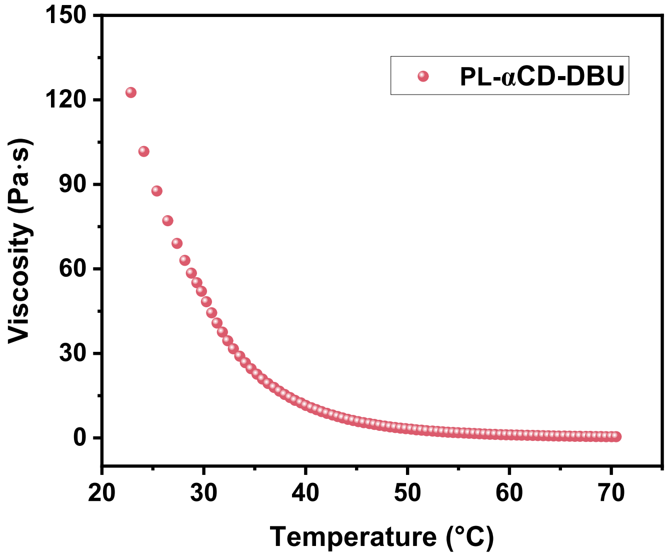

Figure S3. Viscosity-temperature plot of PL-αCD-DBU.

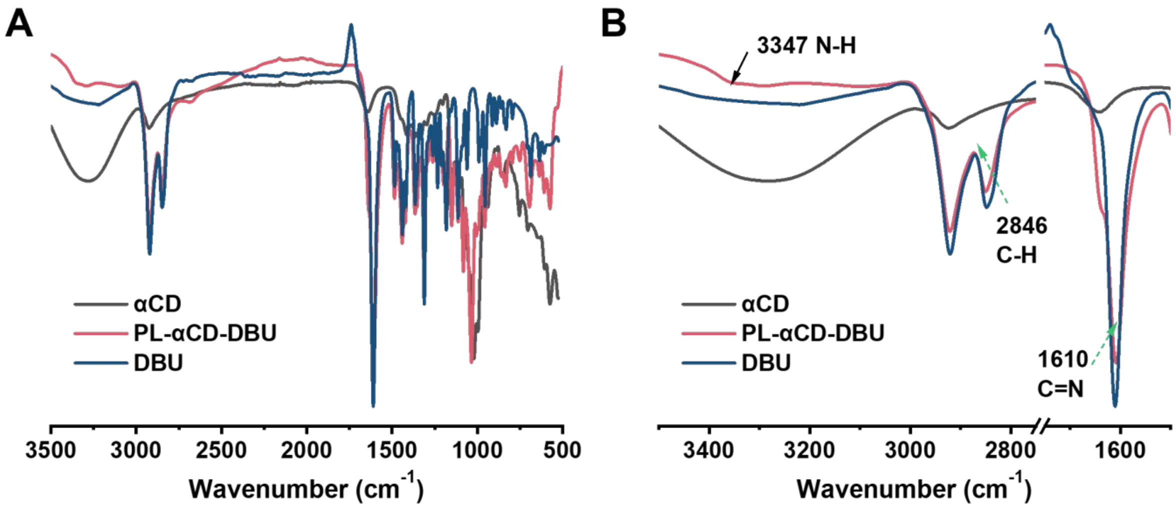

Figure S4. Full-range (A) and zoomed-in (B) FT-IR spectra of αCD, DBU and PL-αCD-DBU.

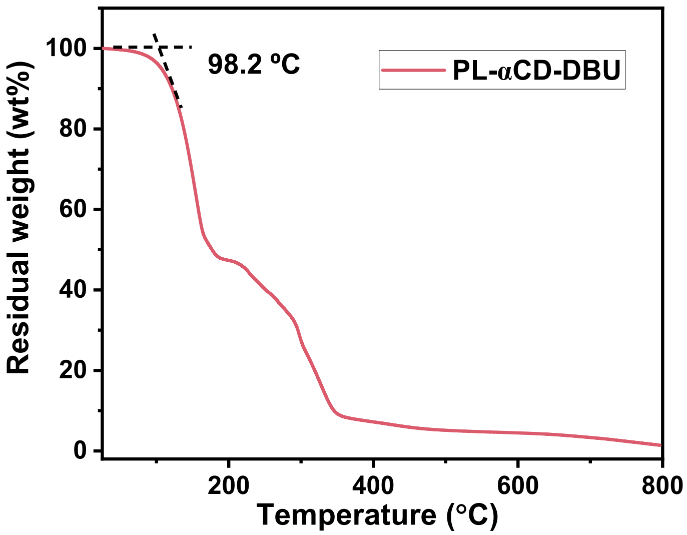

Figure S5. TGA result of PL-αCD-DBU under N_2.

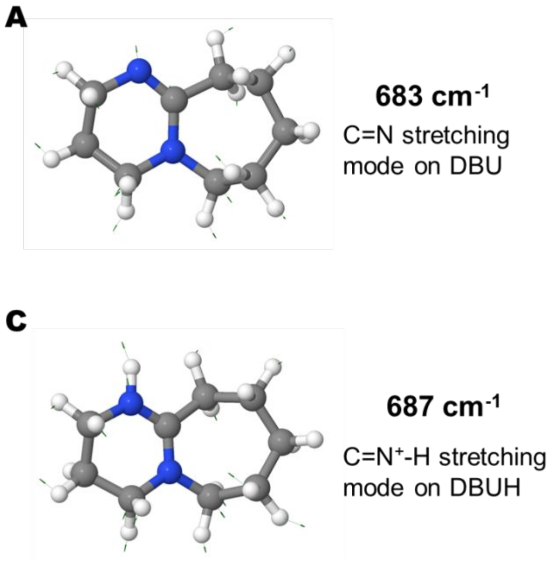

Figure S6. Vibrational modes of DBU and DBUH at different frequencies (visualized with Jmol[15]).

Table S1. Formation energies (ΔE and ΔG, kJ/mol) for DBU–αCD adducts and glucose test-reaction.

Site / Motif	TPSS, ΔE	TPSS, ΔG	TPSSh, ΔE	TPSSh, ΔG
C2 / neutral	−119.6	−43.8	−117.6	−41.8
C3 / neutral	−116.5	−40.3	−114.1	−37.9
C6 / neutral	−92.8	−26.1	−92.6	−25.9
C2 / H-transfer	−113.8	−36.3	−109.4	−31.9
C3 / H-transfer	−109.4	−35.2	−104.8	−30.7
C6 / H-transfer	−92.7	−29.9	−91.0	−28.2
Glucose C6 / neutral	−62.0	+1.4	−60.6	+2.7

1) At the secondary sides (C2/C3), the neutral hydrogen-bonded complexes are more stable than the proton-transfer adducts by approximately 5–10 kJ/mol in ΔG at both theoretical levels.

2) At the primary side (C6), the proton-transfer form is slightly favored, by about 2–4 kJ/mol at both TPSS and TPSSh levels.

3) The absolute formation free energies range from roughly −26 to −44 kJ/mol, indicating a strong association even in the low-dielectric environment.

4) For glucose, only a weak neutral hydrogen bond was obtained, with ΔG ≈ +1–3 kJ/mol, underscoring the role of the macrocyclic α-CD framework in stabilizing stronger adducts. The corresponding proton-transfer minimum could not be found, suggesting that such a structure is either thermodynamically inaccessible or too shallow to represent a stable minimum on the potential-energy surface.

Table S2. Key interatomic distances, Å in optimized DBU–αCD and glucose complexes.

Site / Motif	TPSS/def2-SVPD N···H or N–H	TPSS/def2-SVPD O–H or O···H	TPSSh/def2-SVPD N···H or N–H	TPSSh/def2-SVPD O–H or O···H
C2 / neutral	1.624 (N···H)	1.040 (O–H)	1.647 (N···H)	1.027 (O–H)
C3 / neutral	1.638 (N···H)	1.035 (O–H)	1.660 (N···H)	1.023 (O–H)
C6 / neutral	1.624 (N···H)	1.031 (O–H)	1.642 (N···H)	1.020 (O–H)
C2 / H-transfer	1.105 (N–H)	1.513 (O···H)	1.097 (N–H)	1.517 (O···H)
C3 / H-transfer	1.099 (N–H)	1.533 (O···H)	1.091 (N–H)	1.537 (O···H)
C6 / H-transfer	1.163 (N–H)	1.378 (O···H)	1.148 (N–H)	1.391 (O···H)
Glucose C6 / neutral	1.686 (N···H)	1.020 (O–H)	1.701 (N···H)	1.011 (O–H)

Observed trends:

1) Neutral complexes exhibit short O–H bonds (≈ 1.02–1.04 Å) and hydrogen-bonded N···H distances of 1.62–1.66 Å at all αCD sites (C2, C3, C6).

2) H-transfer adducts display covalent N–H bonds of 1.10–1.16 Å and longer O···H contacts of 1.38–1.53 Å, characteristic of a $[DBUH]^{+}\cdots O^{-}$ ion pair.

3) The C6 H-trasnfer adduct features the shortest O···H contact among the H-transfer structures, consistent with its slight thermodynamic preference at the primary rim.

Our calculations indicate that hydrogen bonding plays an important role in how αCD interacts with DBU. Proton transfer is more favorable at the primary C6 hydroxyls, while the secondary C2 and C3 positions largely retain neutral hydrogen bonds. As a result, the system represents a mix of neutral and partly ionized interactions rather than a complete formation of ion pairs. The slightly unfavorable energetics of the glucose model ($\Delta G \geq 0$) further show that it is the macrocyclic framework of αCD, not simply the acidity of its hydroxyl groups, which provide additional stabilization in these supramolecular complexes.

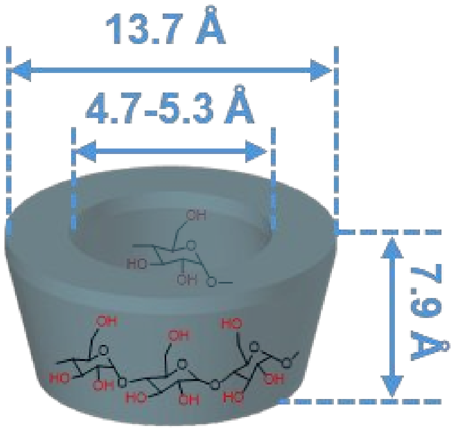

Figure S7. Geometric dimensions of αCD.

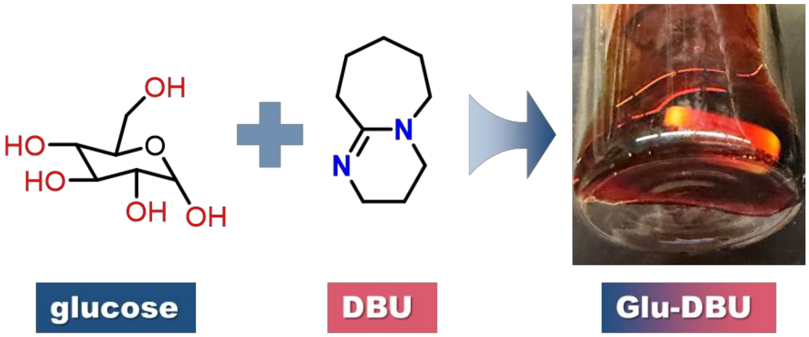

Figure S8. Chemical structures of glucose, and optical image of Glu-DBU.

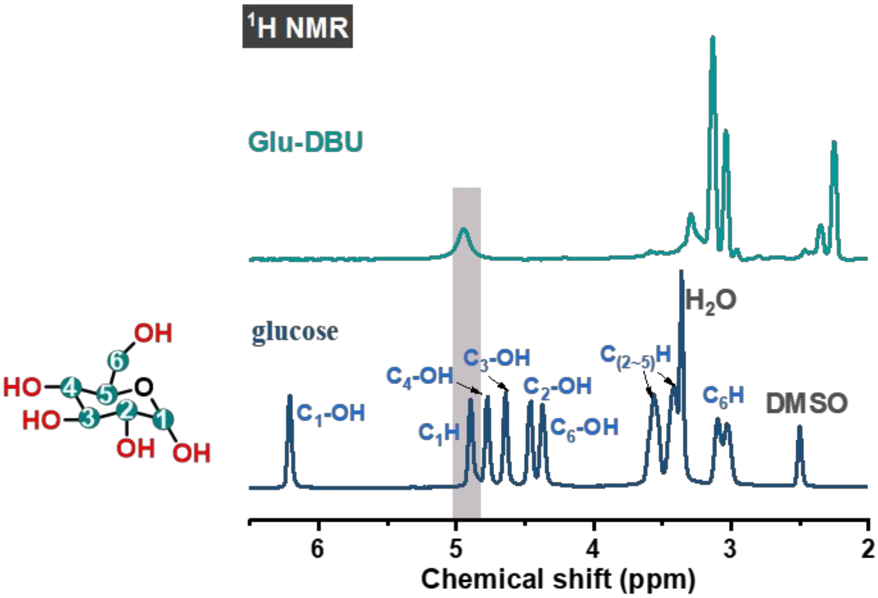

Figure S9. ^{1}H NMR spectra (400 MHz, DMSO-d_6, 298 K) of glucose and Glu-DBU.

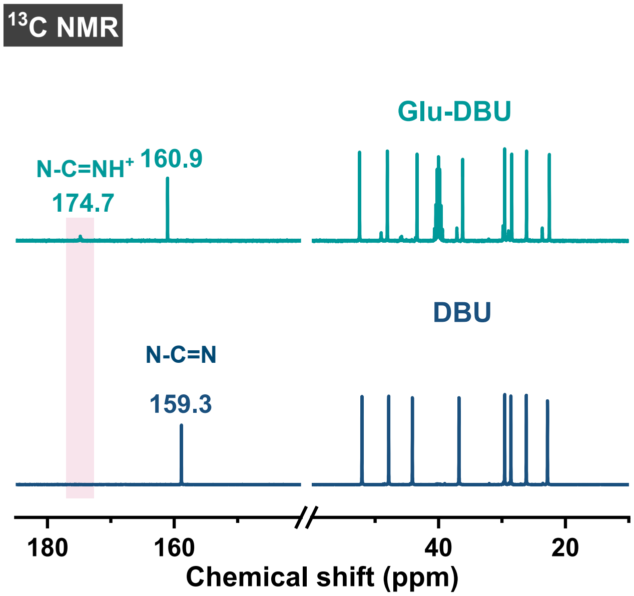

Figure S10. ^{13}C NMR spectra (101 MHz, 298 K) of DBU and Glu-DBU.

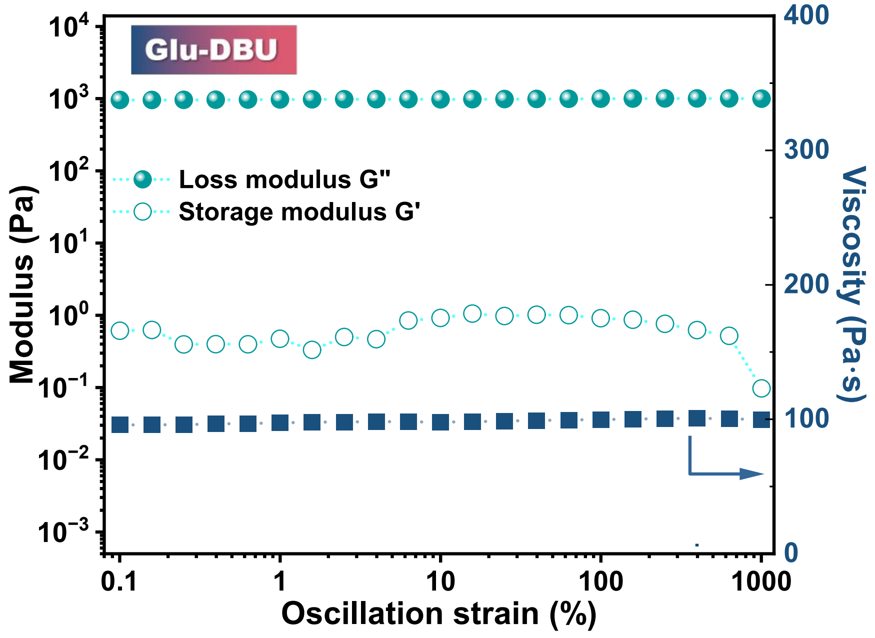

Figure S11. Oscillation-dependent modulus plots and viscosity of Glu-DBU.

Additional discussion: By comparing the oscillation-dependent modulus and viscosity-temperature plots with PL-αCD-DBU (Figure S2 and S3), the Glu-DBU system exhibited only slightly lower viscosity across room temperature and increasing temperatures (96 Pa·s for Glu-DBU *vs* 113 Pa·s for PL-αCD-DBU at room temperature).

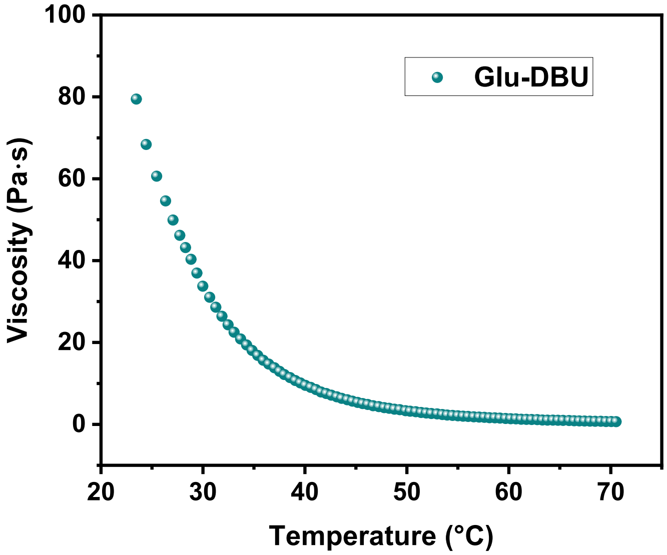

Figure S12. Viscosity-temperature plot of Glu-DBU.

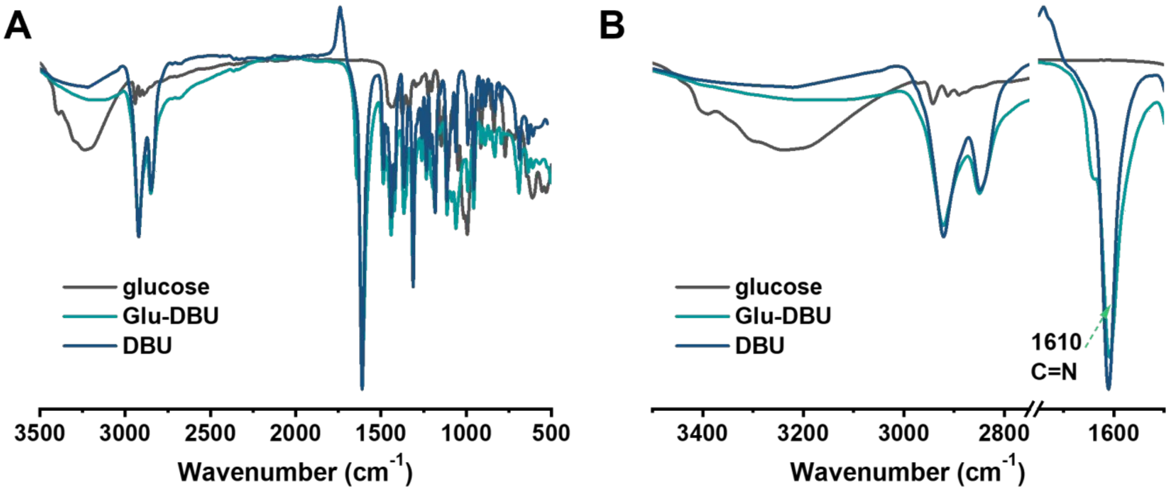

Figure S13. Full-range (A) and zoomed-in (B) FT-IR spectra of glucose, DBU and Glu-DBU.

Additional discussion: the broad O–H stretching band at 3235 cm^{-1} for glucose weakened after mixing with DBU; the C=N stretching near 1610 cm^{-1} showed a red-shift, consistent with the protonated DBU cation formation.

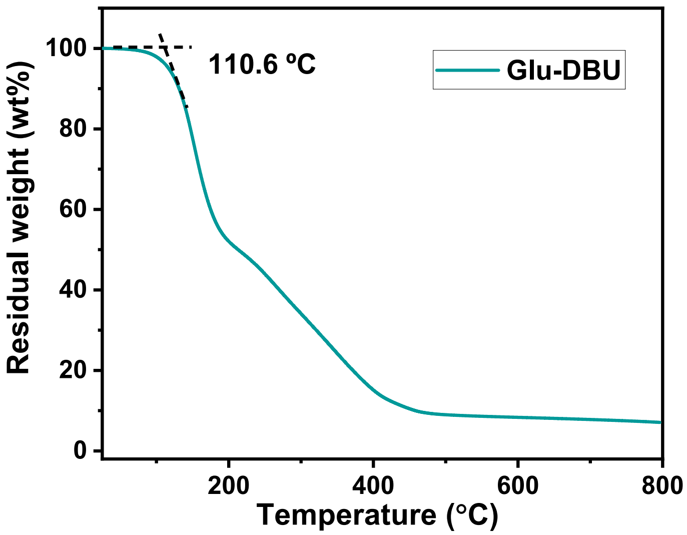

Figure S14. TGA result of Glu-DBU under N_2.

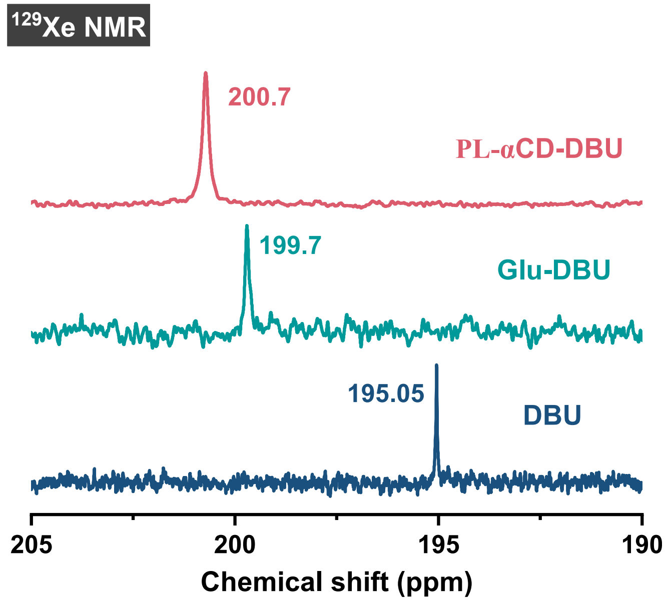

Figure S15. ^{129}Xe NMR spectra for Xe gas in DBU, Glu-DBU and PL-αCD-DBU.

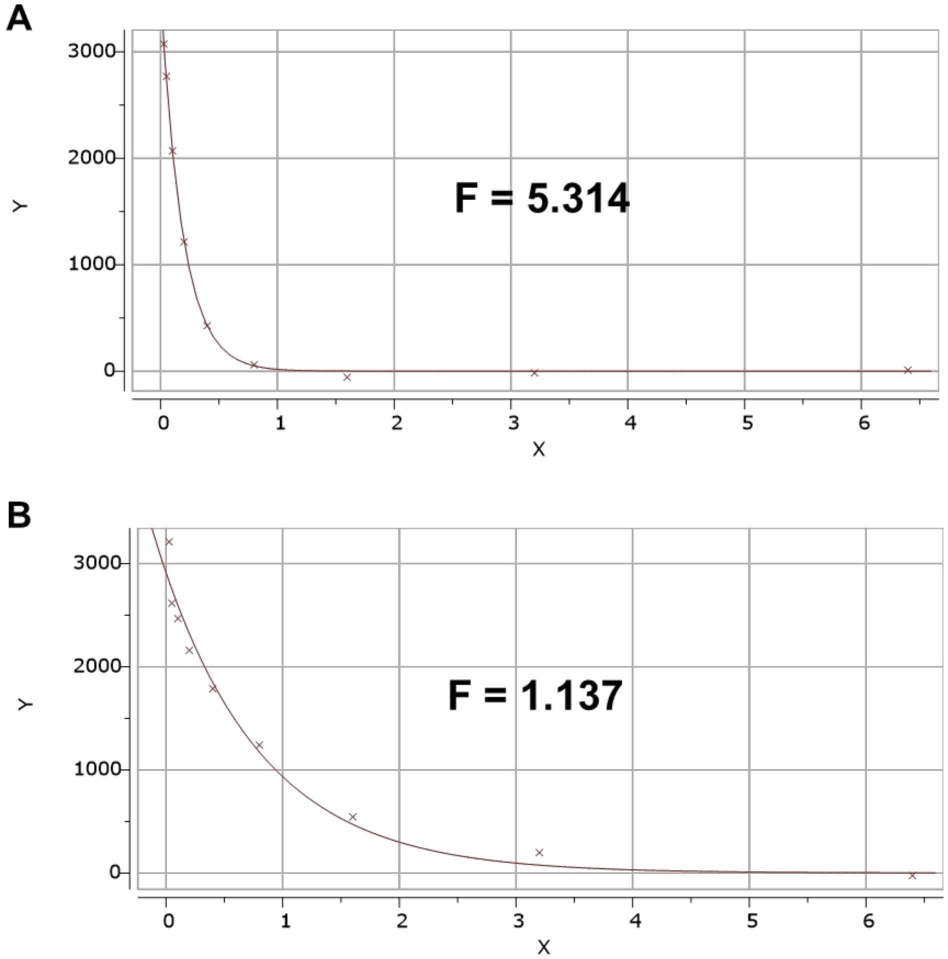

Figure S16. Non-linear fitting of the peaks at -80.5 (A) and -83.6 (B) ppm from stack plot of ^{19}F NMR spectra of PFH@PL-αCD-DBU, using MestReNova software.

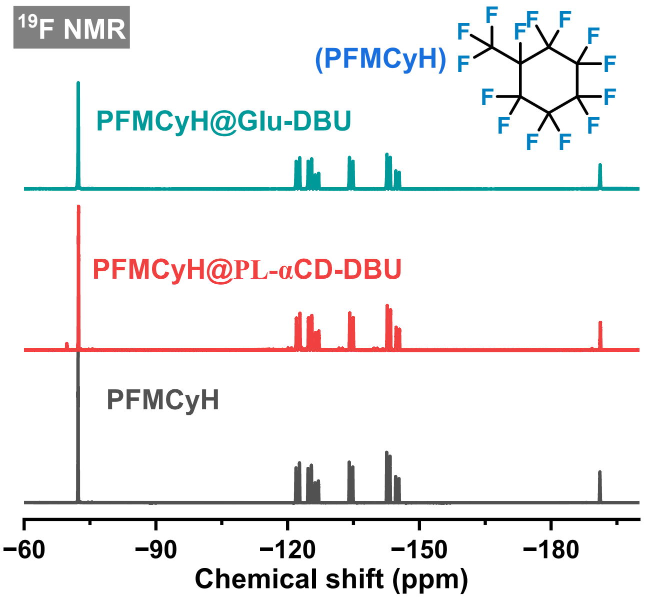

Figure S17. Solvent-free ^{19}F NMR spectra of PFMCyH, PFMCyH@PL-αCD-DBU, and PFMCyH@Glu-DBU.

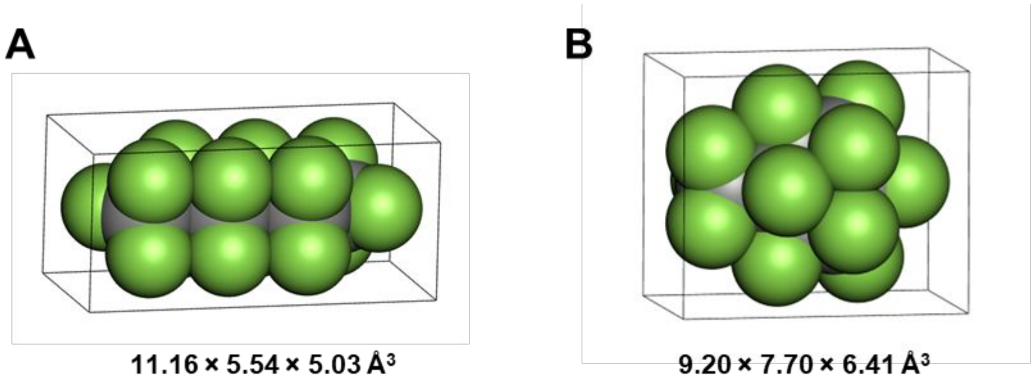

Figure S18. Space-filling models of PFH (A) and PFMCyH (B) showing the molecular dimensions.

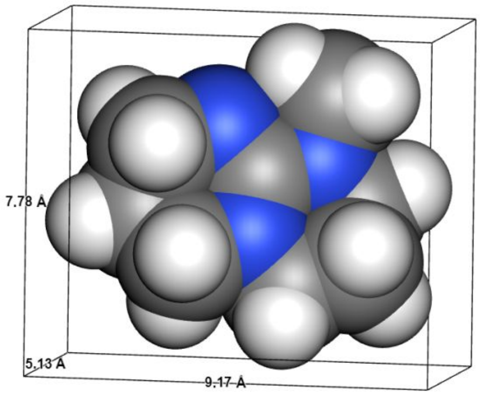

Figure S19. Space-filling model of MTBD showing the molecular dimensions (9.17 × 5.13 × 7.78 Å^3).

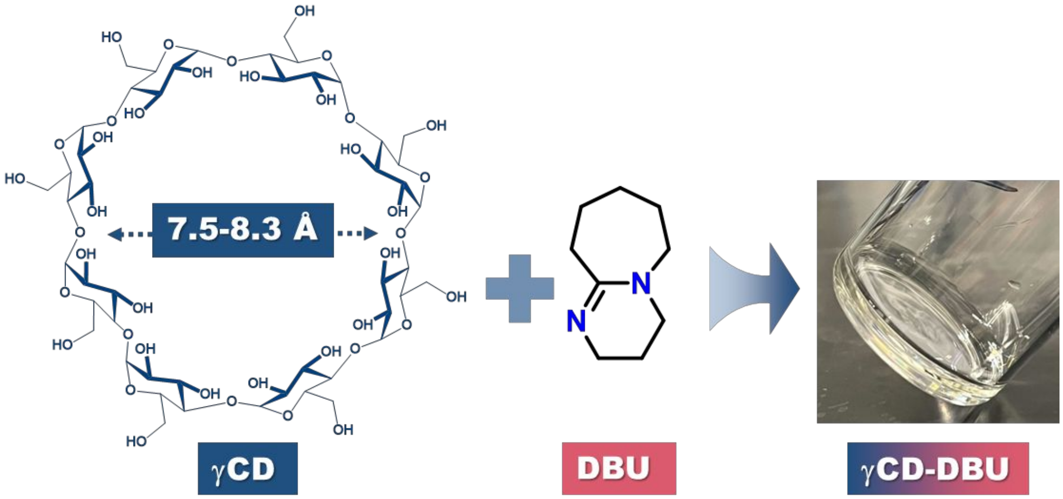

Figure S20. Chemical structures of γCD, and optical image of γCD-DBU.

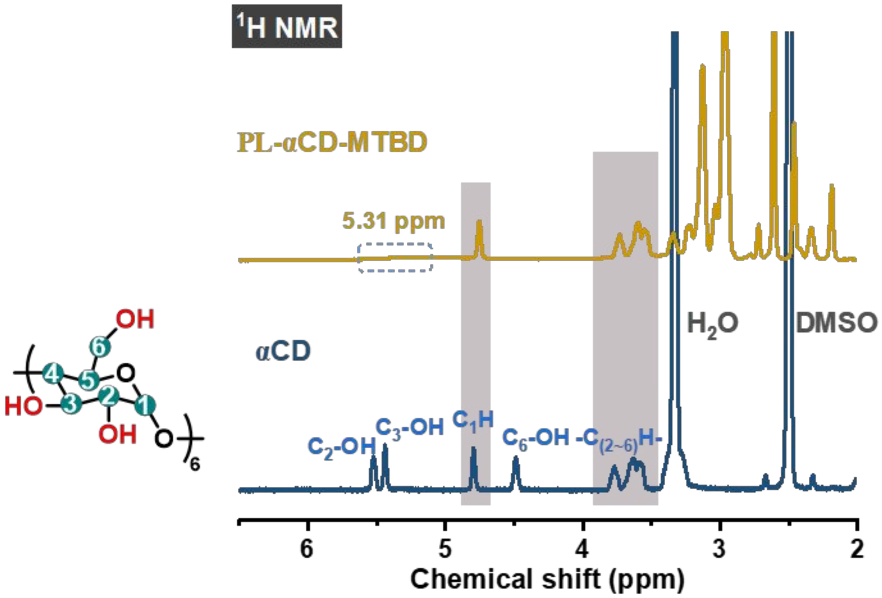

Figure S21. ^{1}H NMR spectra (400 MHz, DMSO-d_6, 298 K) of αCD and PL-αCD-MTBD.

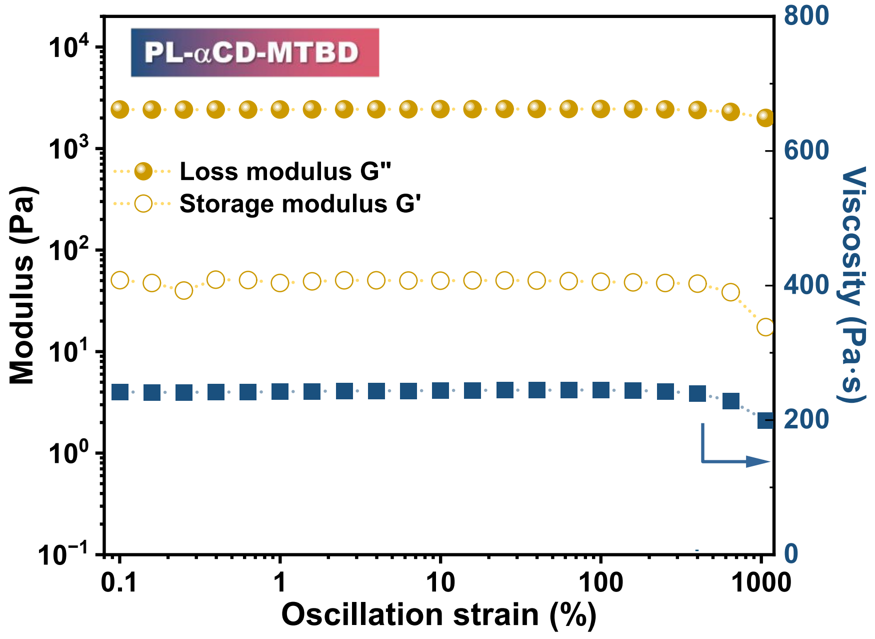

Figure S22. Oscillation-dependent modulus plots and viscosity of PL-αCD-MTBD.

Additional discussion: Comparison of the oscillation-dependent modulus plots across different systems reveals that, when DBU is used as the basic motif, increasing the number of building units results in a slight increase in viscosity for the βCD- and γCD-based systems relative to the αCD-based system (187 Pa·s for PL-βCD-DBU *vs* 141 Pa·s for γCD-DBU *vs* 113 Pa·s for PL-αCD-DBU at room temperature). Substituting DBU with MTBD, however, leads to a significantly greater increase in viscosity (244 Pa·s for PL-αCD-MTBD *vs* 113 Pa·s for PL-αCD-DBU at room temperature). Furthermore, all systems display a pronounced time-dependent decrease in viscosity.

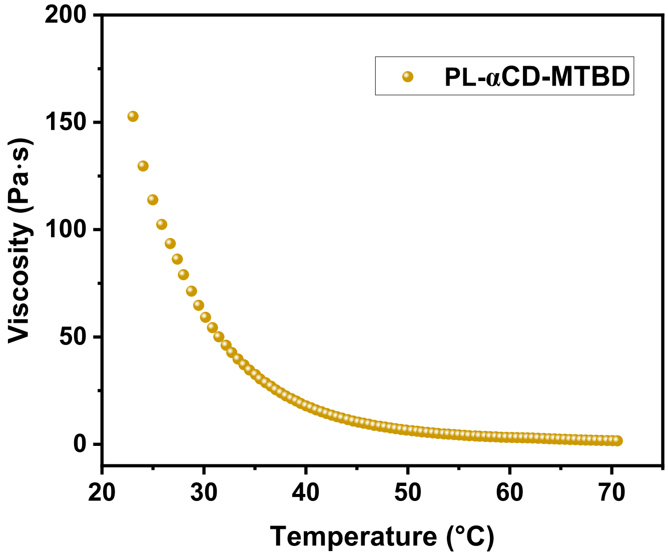

Figure S23. Viscosity-temperature plot of PL-αCD-MTBD.

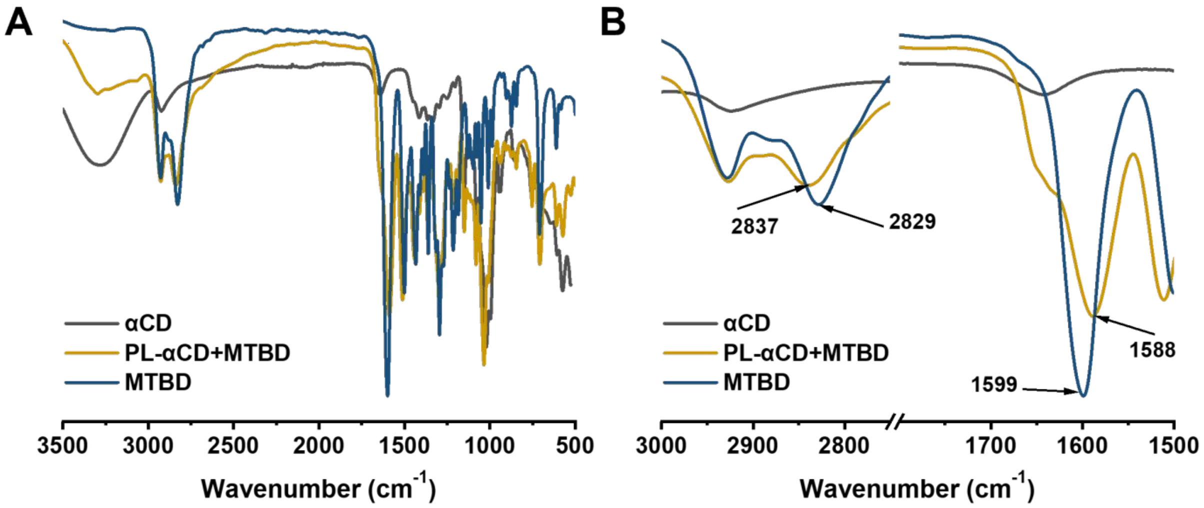

Figure S24. Full-range (A) and zoomed-in (B) FT-IR spectra of αCD, MTBD and PL-αCD-MTBD.

Additional discussion: the broad O–H stretching band at 3288 cm^{-1} for αCD weakened after mixing with MTBD; the methylene C–H stretching of MTBD exhibited a blue-shift from 2829 to 2837 cm^{-1}; the C=N stretching showed a red-shift from 1599 to 1588 cm^{-1}, consistent with the protonated MTBD cation formation.

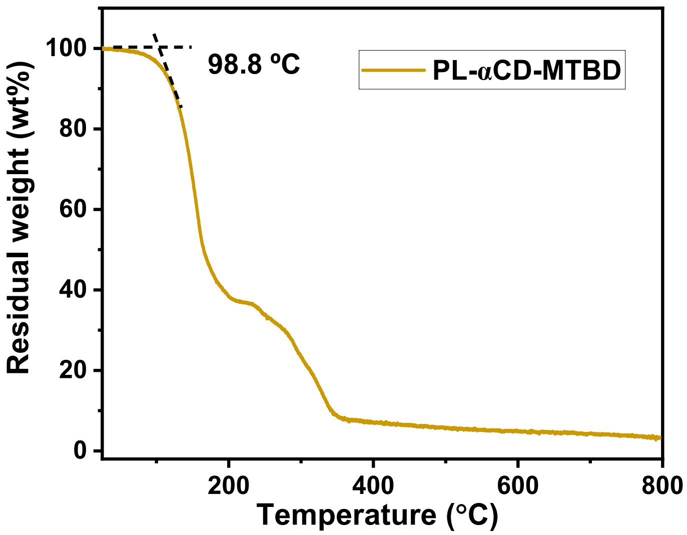

Figure S25. TGA result of PL-αCD- MTBD under N_2.

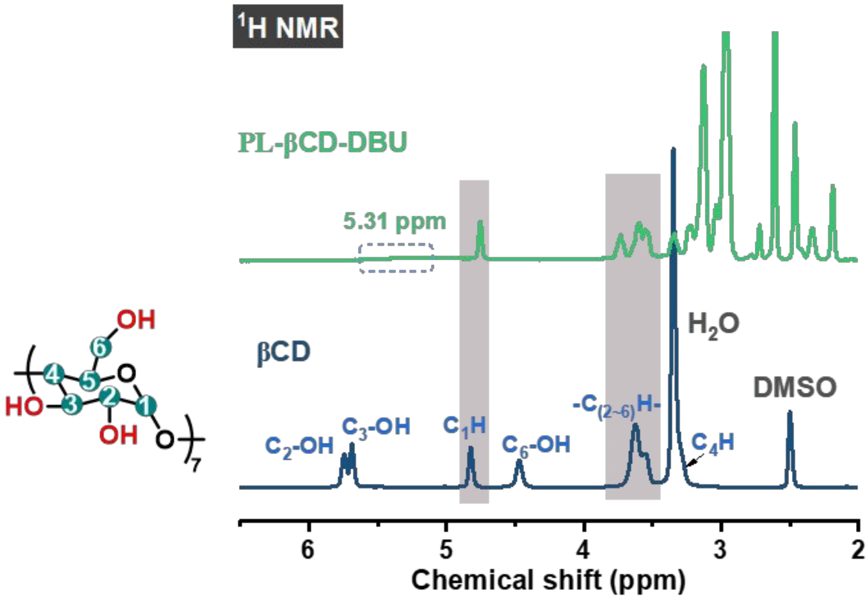

Figure S26. ^{1}H NMR spectra (400 MHz, DMSO-d_6, 298 K) of βCD and PL-βCD-DBU.

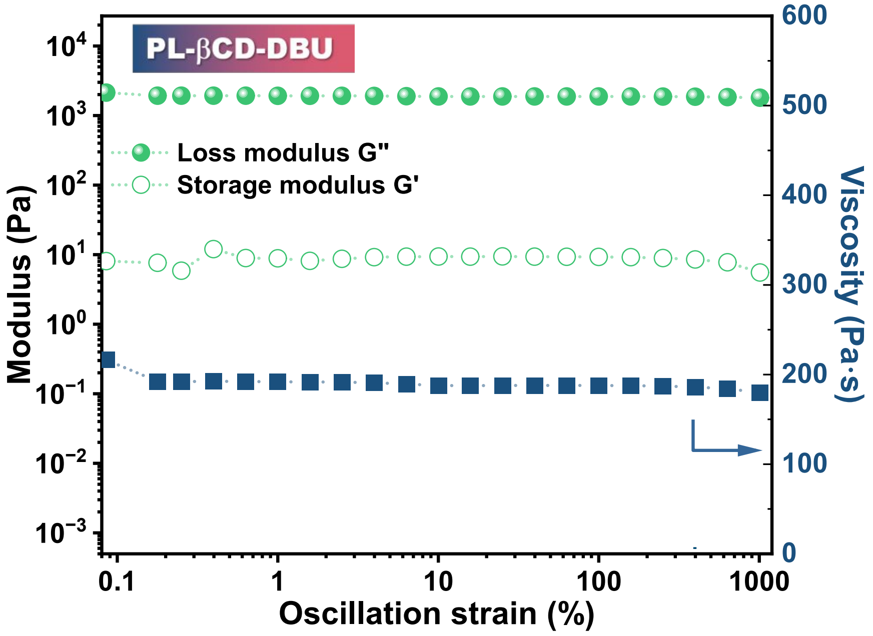

Figure S27. Oscillation-dependent modulus plots and viscosity of PL-βCD-DBU.

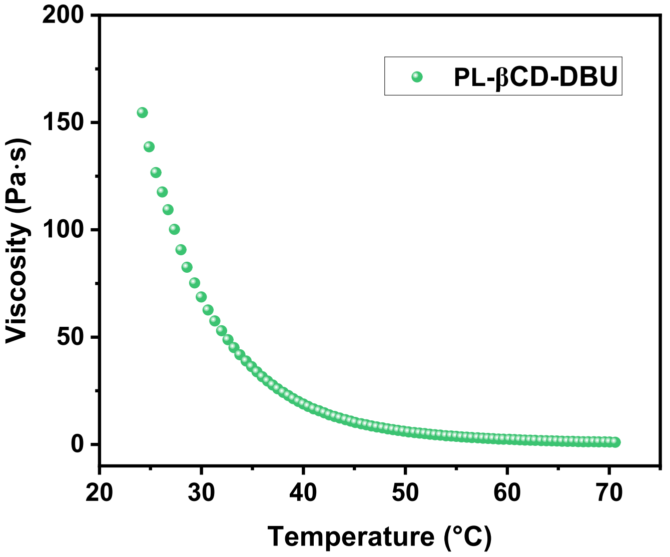

Figure S28. Viscosity-temperature plot of PL-βCD-DBU.

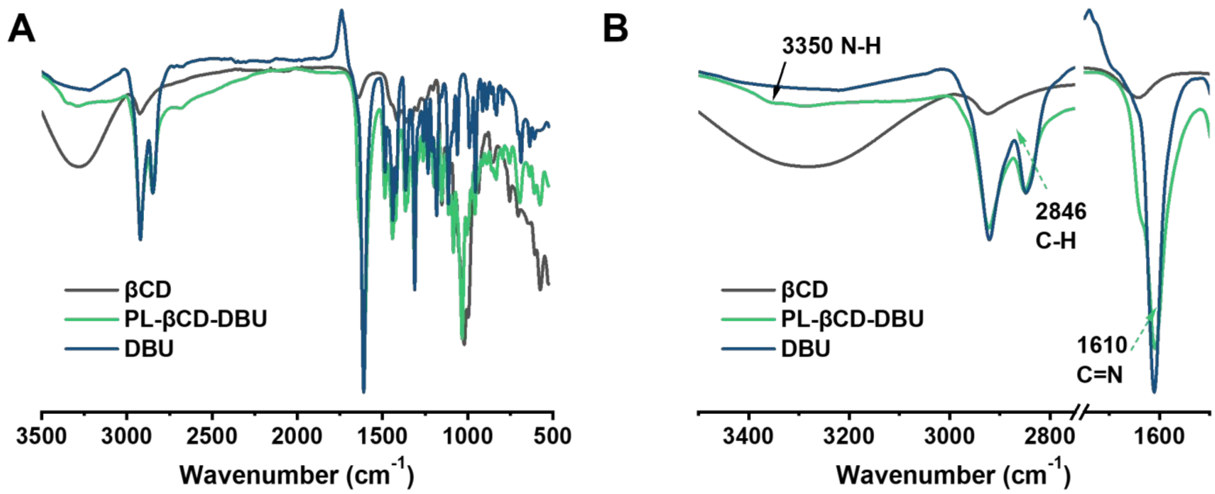

Figure S29. Full-range (A) and zoomed-in (B) FT-IR spectra of βCD, DBU and PL-βCD-DBU.

Additional discussion: the broad O–H stretching band at 3281 cm^{-1} for βCD progressively weakened after mixing with DBU, accompanied by the appearance of an N–H stretching band at 3350 cm^{-1}; the methylene C–H stretching of DBU at 2846 cm^{-1} exhibited a blue-shift; the C=N stretching near 1610 cm^{-1} showed a red-shift, consistent with the protonated DBU cation formation.

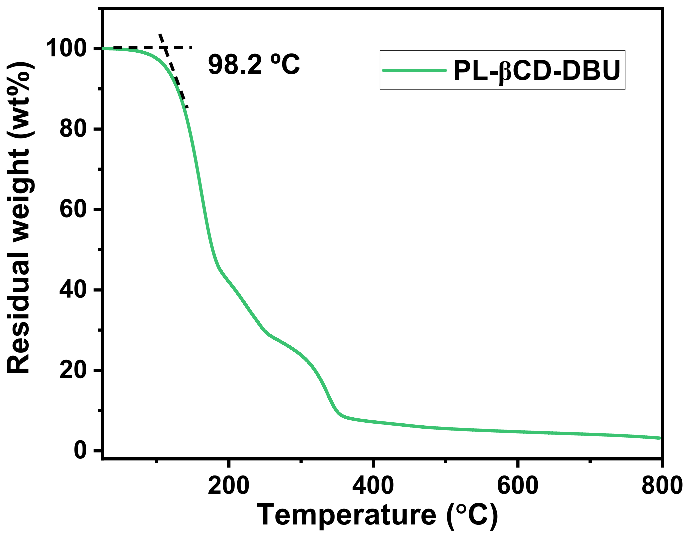

Figure S30. TGA result of PL-βCD-DBU under N_2.

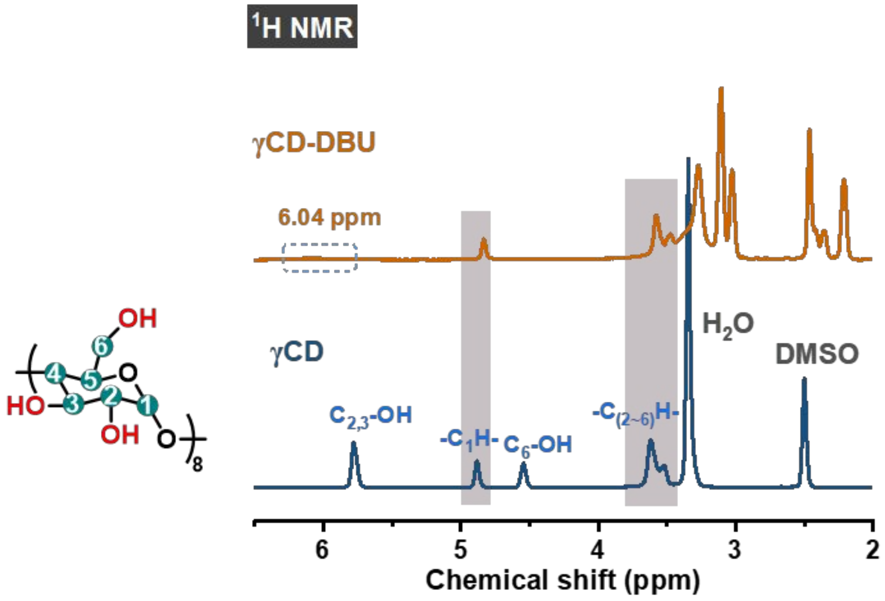

Figure S31. ¹H NMR spectra (400 MHz, DMSO-d_6, 298 K) of γCD and γCD-DBU.

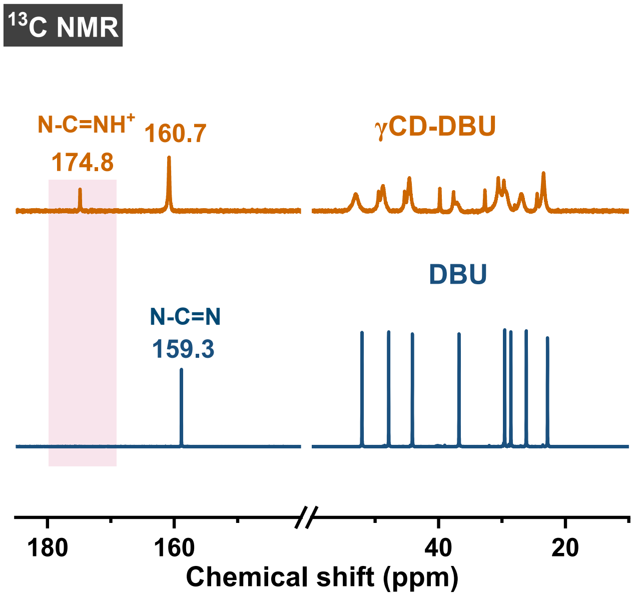

Figure S32. ^{13}C NMR spectra (400 MHz, DMSO-d_6, 298 K) of DBU and γCD-DBU.

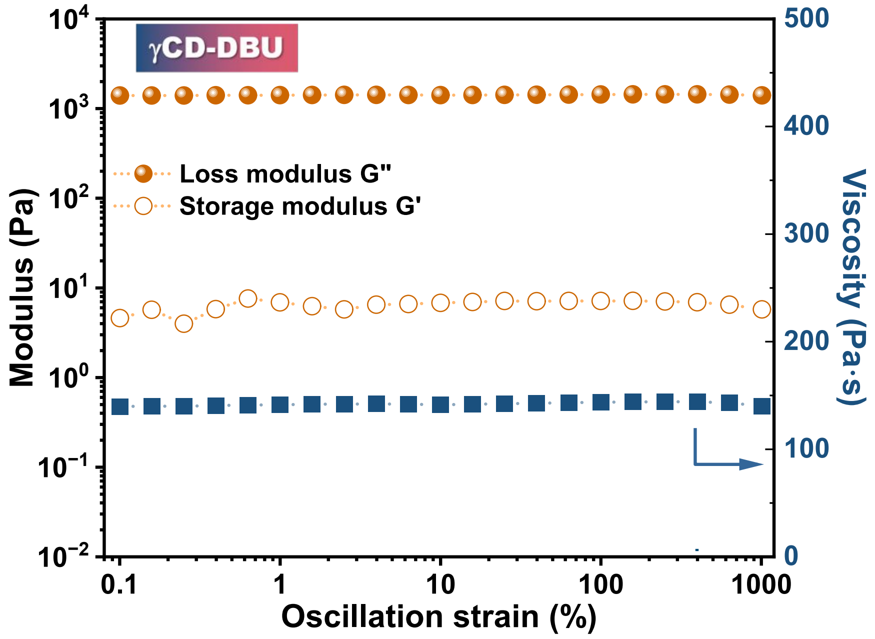

Figure S33. Oscillation-dependent modulus plots and viscosity of γCD-DBU.

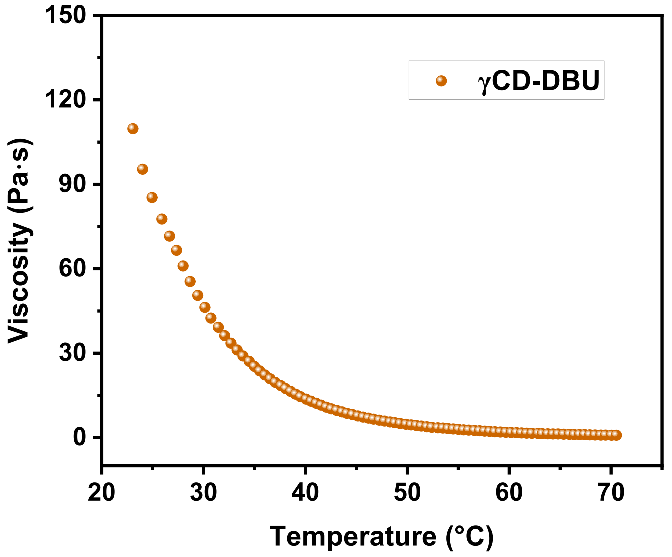

Figure S34. Viscosity-temperature plot of γCD-DBU.

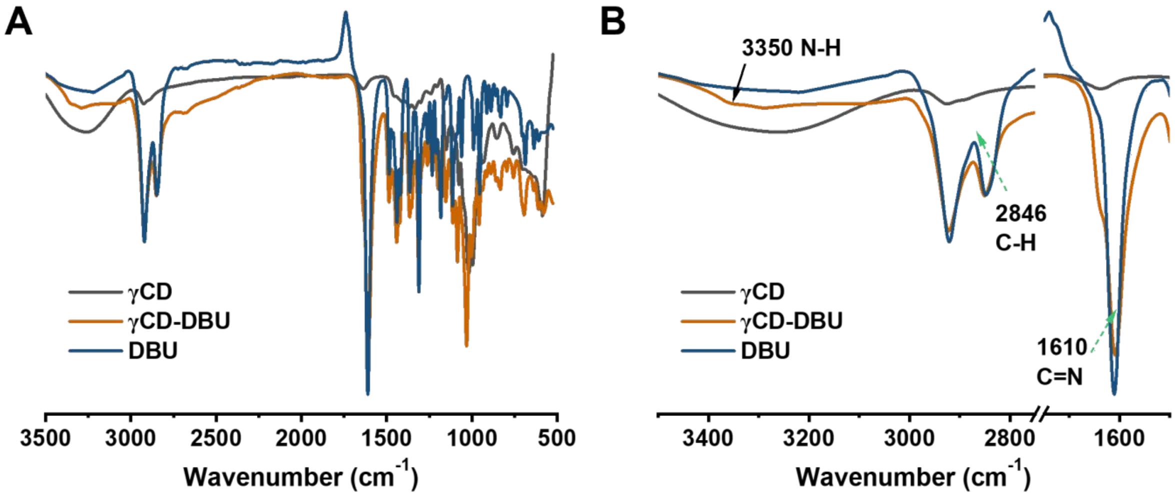

Figure S35. Full-range (A) and zoomed-in (B) FT-IR spectra of γCD, DBU and γCD-DBU.

Additional discussion: the broad O–H stretching band at 3261 cm^{-1} for γCD weakened after mixing with DBU, accompanied by the appearance of an N–H stretching band at 3350 cm^{-1}; the methylene C–H stretching of DBU at 2846 cm^{-1} exhibited a blue-shift; the C=N stretching near 1610 cm^{-1} showed a red-shift, consistent with the protonated DBU cation formation.

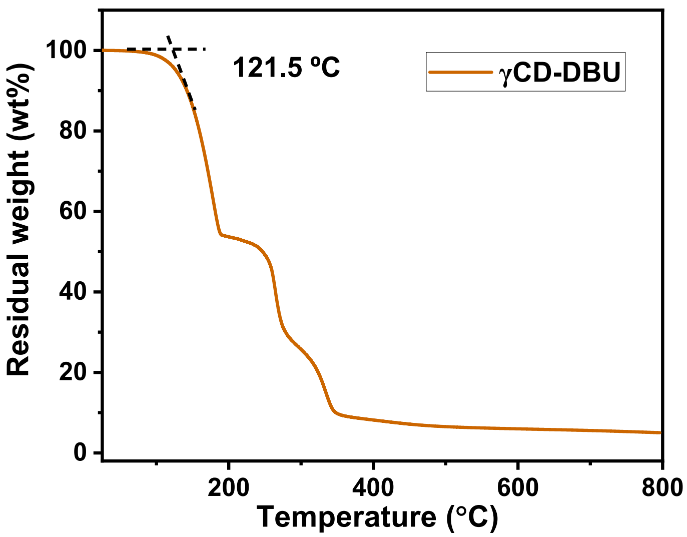

Figure S36. TGA result of γCD-DBU under N_2.

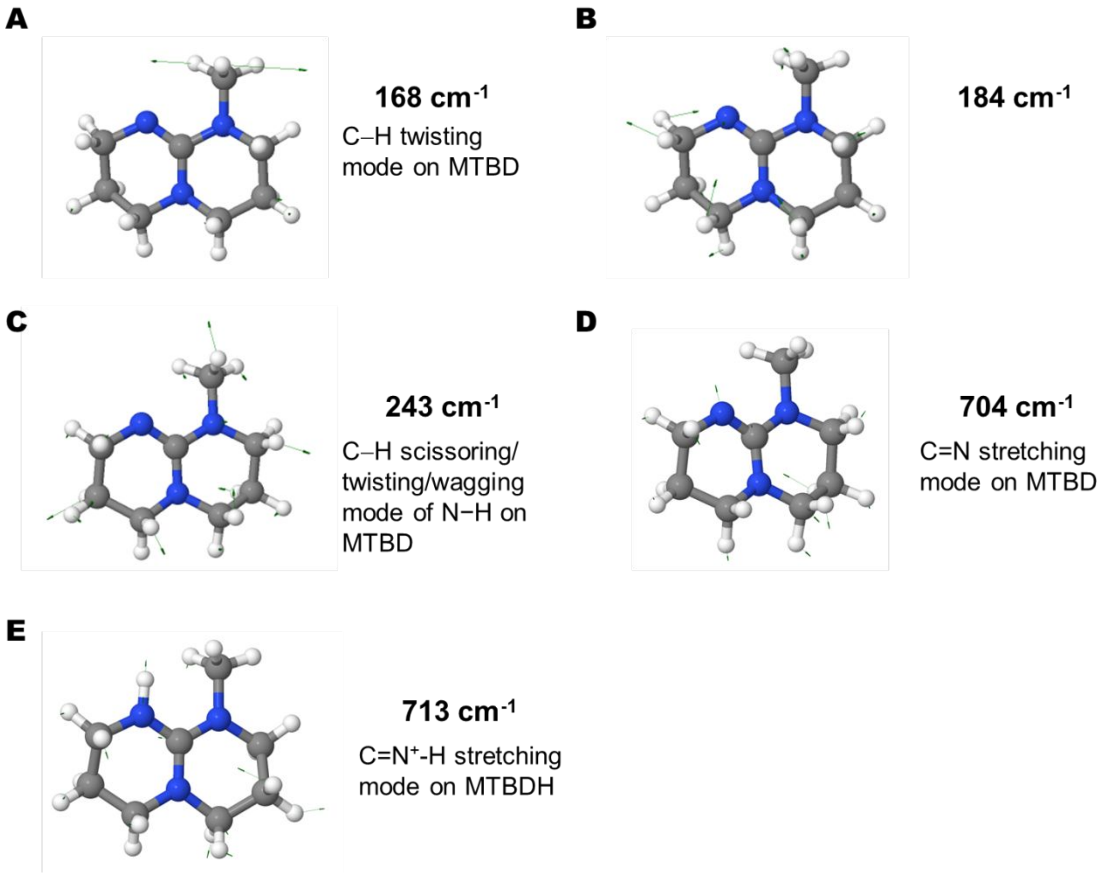

Figure S37. Vibrational modes of MTBD and protonated MTBD (MTBDH) at different frequencies.

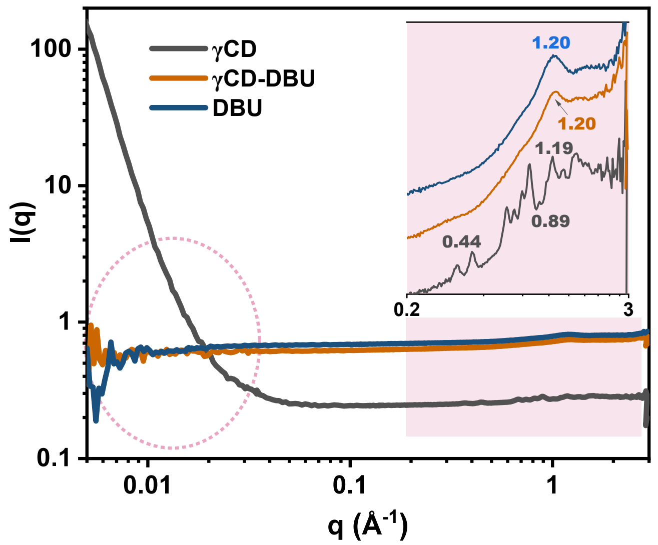

Figure S38. EQ-SANS profiles of γCD, DBU, and γCD-DBU.

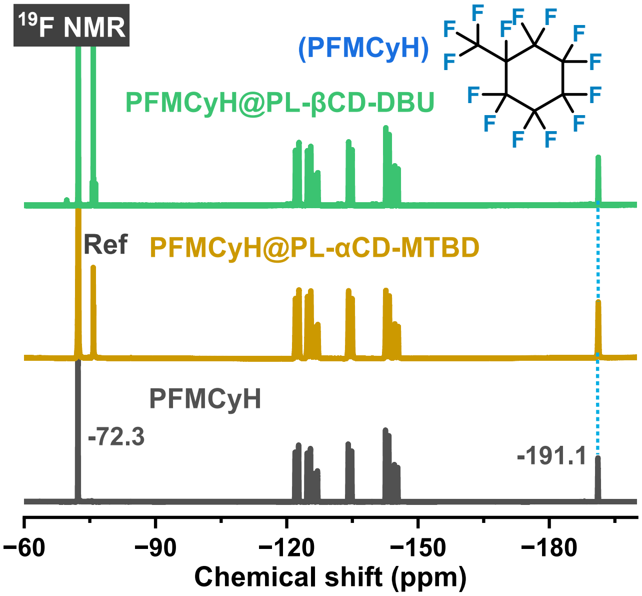

Figure S39. ^{19}F NMR spectra of PFMCyH, PFMCyH@PL-αCD-MTBD and PFMCyH@PL-βCD-DBU.

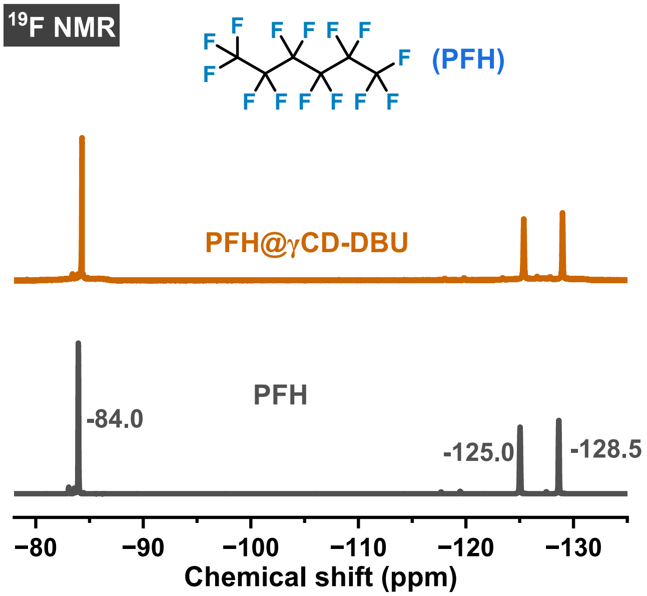

Figure S40. ^{19}F NMR spectra of PFH and PFH@γCD-DBU.

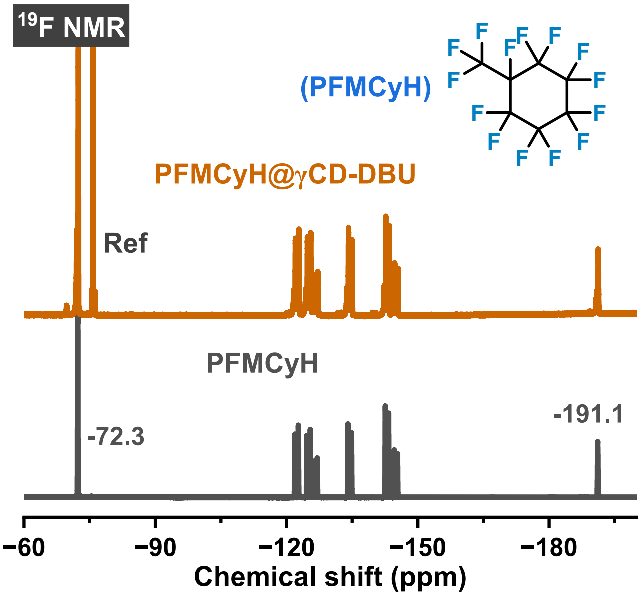

Figure S41. ^{19}F NMR spectra of PFMCyH and PFMCyH@γCD-DBU.

3. References

(1) Frisch, M.; Trucks, G.; Schlegel, H.; Scuseria, G.; Robb, M.; Cheeseman, J.; Scalmani, G.; Barone, V.; Mennucci, B.; Petersson, G. Gaussian 09, Revision E. 01, 2013, Gaussian. *Inc., Wallingford CT* **2016**.

(2) Cheng, Y.; Daemen, L. L.; Kolesnikov, A. I.; Ramirez-Cuesta, A. J. Simulation of inelastic neutron scattering spectra using OCLIMAX. *J. Chem. Theory Comput.* **2019**, *15* (3), 1974-1982.

(3) Heller, W. T.; Hetrick, J.; Bilheux, J.; Calvo, J. M. B.; Chen, W.-R.; DeBeer-Schmitt, L.; Do, C.; Doucet, M.; Fitzsimmons, M. R.; Godoy, W. F.; Granroth, G. E.; Hahn, S.; He, L.; Islam, F.; Lin, J.; Littrell, K. C.; McDonnell, M.; McGaha, J.; Peterson, P. F.; Pingali, S. V.; Qian, S.; Savici, A. T.; Shang, Y.; Stanley, C. B.; Urban, V. S.; Whitfield, R. E.; Zhang, C.; Zhou, W.; Billings, J. J.; Cuneo, M. J.; Leal, R. M. F.; Wang, T.; Wu, B. drtsans: The data reduction toolkit for small-angle neutron scattering at Oak Ridge National Laboratory. *SoftwareX* **2022**, *19*, 101101.

(4) Heller, W. T.; Cuneo, M.; Debeer-Schmitt, L.; Do, C.; He, L.; Heroux, L.; Littrell, K.; Pingali, S. V.; Qian, S.; Stanley, C.; Urban, V. S.; Wu, B.; Bras, W. The suite of small-angle neutron scattering instruments at Oak Ridge National Laboratory. *J. Appl. Cryst.* **2018**, *51* (2), 242-248.

(5) Tao, J.; Perdew, J. P.; Staroverov, V. N.; Scuseria, G. E. Climbing the density functional ladder: Nonempirical meta–generalized gradient approximation designed for molecules and solids. *Phys. Rev. Lett.* **2003**, *91* (14), 146401.

(6) Weigend, F.; Ahlrichs, R. Balanced basis sets of split valence, triple zeta valence and quadruple zeta valence quality for H to Rn: Design and assessment of accuracy. *Phys. Chem. Chem. Phys.* **2005**, *7* (18), 3297-3305.

(7) Rappoport, D.; Furche, F. Property-optimized Gaussian basis sets for molecular response calculations. *J. Chem. Phys.* **2010**, *133* (13).

(8) Hellweg, A.; Rappoport, D. Development of new auxiliary basis functions of the Karlsruhe segmented contracted basis sets including diffuse basis functions (def2-SVPD, def2-TZVPPD, and def2-QVPPD) for RI-MP2 and RI-CC calculations. *Phys. Chem. Chem. Phys.* **2015**, *17* (2), 1010-1017.

(9) Caldeweyher, E.; Ehlert, S.; Hansen, A.; Neugebauer, H.; Spicher, S.; Bannwarth, C.; Grimme, S. A generally applicable atomic-charge dependent London dispersion correction. *J. Chem. Phys.* **2019**, *150* (15).

(10) Staroverov, V.; Scuseria, G.; Tao, J.; Perdew, J. Deviations of calculated properties from experiment for species of the G3/99, T-96R, and T-82F test sets. Properties of hydrogen-bonded complexes. *J. Chem. Phys.* **2003**, *119*, 12129-12137.

(11) Cossi, M.; Rega, N.; Scalmani, G.; Barone, V. Energies, structures, and electronic properties of molecules in solution with the C-PCM solvation model. *J. Comput. Chem.* **2003**, *24* (6), 669-681.

(12) Neese, F.; Wennmohs, F.; Becker, U.; Riplinger, C. The ORCA quantum chemistry program package. *J. Chem. Phys.* **2020**, *152* (22).

(13) Abraham, M. J.; Murtola, T.; Schulz, R.; Páll, S.; Smith, J. C.; Hess, B.; Lindahl, E. GROMACS: High performance molecular simulations through multi-level parallelism from laptops to supercomputers. *SoftwareX* **2015**, *1*, 19-25.

(14) Wang, J.; Wolf, R. M.; Caldwell, J. W.; Kollman, P. A.; Case, D. A. Development and testing of a general amber force field. *J. Comput. Chem.* **2004**, *25* (9), 1157-1174.

(15) Humphrey, W.; Dalke, A.; Schulten, K. VMD: visual molecular dynamics. *J. Mol. Graphics* **1996**, *14* (1), 33-38.
